# Giantin mediates Golgi localization of Gal3-O-sulfotransferases and affects salivary mucin sulfation in patients with Sjögren’s disease

**DOI:** 10.1172/jci.insight.171585

**Published:** 2024-11-22

**Authors:** Matilde Nuñez, Patricia Carvajal, Sergio Aguilera, María-José Barrera, Soledad Matus, Alicia Couto, Malena Landoni, Gaelle Boncompain, Sergio González, Claudio Molina, Karina Pino, Sebastián Indo, Lourdes Figueroa, María-Julieta González, Isabel Castro

**Affiliations:** 1 Instituto de Ciencias Biomédicas, Facultad de Medicina, Universidad de Chile, Santiago, Chile.; 2 Clínica INDISA, Santiago, Chile.; 3 Facultad de Odontología y Ciencias de la Rehabilitación, Universidad San Sebastián, Santiago, Chile.; 4 Centro Científico y Tecnológico de Excelencia Ciencia & Vida, Fundación Ciencia & Vida, Facultad de Medicina y Ciencia, Universidad San Sebastian, Santiago, Chile.; 5 Departamento de Química Orgánica, Facultad de Ciencias Exactas y Naturales, Universidad de Buenos Aires, Consejo Nacional de Investigaciones Científicas y Técnicas, Centro de Investigación en Hidratos de Carbono, Buenos Aires, Argentina.; 6 Dynamics of Intracellular Organization Laboratory, Institut Curie, PSL Research University, Sorbonne Université, Centre National de la Recherche Scientifique, UMR 144, Paris, France.; 7 Escuela de Odontología, Facultad de Medicina y Ciencias de la Salud, Universidad Mayor, Santiago, Chile.; 8 Departamento de Tecnología Médica, Facultad de Medicina, Universidad de Chile, Santiago, Chile.

**Keywords:** Autoimmunity, Cell biology, Autoimmune diseases, Glycobiology, Protein traffic

## Abstract

Sjögren’s disease is a chronic autoimmune disease characterized by symptoms of oral and ocular dryness and extraglandular manifestations. Mouth dryness is not only due to reduced saliva volume, but also to alterations in the quality of salivary mucins in patients with Sjögren’s disease. Mucins play a leading role in mucosa hydration and protection, where sulfated and sialylated oligosaccharides retain water molecules at the epithelial surface. The correct localization of glycosyltransferases and sulfotransferases within the Golgi apparatus determines adequate O-glycosylation and sulfation of mucins, which depends on specific golgins that tether enzyme-bearing vesicles. Here, we show that a golgin called Giantin was mislocalized in salivary glands from patients with Sjögren’s disease and formed protein complexes with Gal3-O-sulfotransferases (Gal3STs), which changed their localization in Giantin-knockout and -knockdown cells. Our results suggest that Giantin could tether Gal3ST-bearing vesicles and that its altered localization could affect Gal3ST activity, explaining the decreased sulfation of MUC5B observed in salivary glands from patients with Sjögren’s disease.

## Abstract 2

Giantin mediates the Golgi localization of Gal3-O-sulfotransferases and Giantin mislocalization is implicated in the altered sulfation of salivary mucins in patients with Sjögren’s disease

## Introduction

Sjögren’s disease (SjD) is the second-most common autoimmune disease, after rheumatoid arthritis. SjD is a systemic, chronic, and inflammatory exocrinopathy, characterized by reduced secretory function, mainly of the lacrimal and salivary glands (1). Its main symptoms include eye and mouth dryness, fatigue, musculoskeletal pain, and swelling of the major salivary glands. The affected may have gastrointestinal, renal, pulmonary, dermatological, neurological, and hematological manifestations, with an increased risk of developing non–Hodgkin’s B cell lymphoma (1). In some cases, the gradual and progressive damage and dysfunction of the exocrine glands result in whole-body dryness (the sicca syndrome) (2). Xerostomia is the subjective sensation of dry mouth, and many patients with SjD experience this symptom despite having a normal salivary flow, suggesting that the dryness is not merely a consequence of reduced saliva volume (3–5). Mucins are high-molecular-weight glycoproteins containing oligosaccharide side chains attached to a central peptide. Sulfate esters and sialylated derivatives turn mucins into hydrophilic polyanionic polymers capable of retaining large amounts of water within the epithelial surface, aiding in the maintenance of mucosal hydration (6, 7). Individuals with and without xerostomia do not seem to show differential concentrations of the mucins in the residual saliva on the oral mucosa after swallowing (8). This evidence suggests that qualitative alterations in the saliva’s rheological properties in patients with SjD are the main cause of mouth dryness and complications of the oral cavity, such as burning, mucosal ulceration, swallowing difficulties, and bacterial and mycotic infections (5, 8). The protein backbone of mucins, known as apomucin, is synthesized in the rough endoplasmic reticulum (RER) and contains a domain comprising repeated sequences rich in serine and threonine amino acids (6, 9). O-glycosylation is a compartmentalized and sequential metabolic pathway that takes place in the Golgi apparatus and is facilitated by a group of enzymes known as glycosyltransferases. O-glycosylation is initiated by N-acetyl galactosaminyl transferases (ppGalNAcT), which transfer N-acetylgalactosamine (GalNAc) to the serine or threonine residues of the mucin protein backbone (10). The product of this reaction, GalNAc-serine/threonine, can be extended by other glycosyltransferases and modified by sulfotransferases to generate a broad spectrum of oligosaccharides (11, 12). Glycosyltransferases are localized in different compartments of the Golgi apparatus according to their enzymatic activity (13). Enzymes that act during the first steps of O-glycosylation are distributed in *cis*-Golgi, and the enzymes that terminally modify the oligosaccharides are concentrated in *trans*-Golgi (14). In the secretory pathway, vesicles are recruited by tethering factors to ensure the specificity and efficiency of their fusion with the target membrane (15). Golgins are tethering factors that capture enzyme-bearing vesicles, allowing enzymes to arrive at their correct destination within the Golgi compartments (16, 17). In pancreatic cancer–derived cells (Panc-1), transport vesicles carrying the C2GnT enzyme are recruited to the Golgi apparatus exclusively by Giantin, whereas vesicles carrying the C1GalT enzyme are recruited by the GM130 golgin (17). Silencing Giantin in Panc-1 cells interferes with the localization of C2GnT in the Golgi apparatus, and the localization of C1GalT is also disrupted when GM130 is silenced (17).

Analysis of mucin glycosylation levels in the residual saliva of patients with xerostomia showed a significantly decreased oligosaccharide/apomucin ratio, suggesting altered O-glycosylation of mucins (18). In addition, patients with SjD show decreased levels of MUC7-associated sialylated oligosaccharides in the residual saliva (19) and a decreased Sialyl-Lewis^a^/MUC7 ratio in labial salivary glands (LSGs) (20). Consistent with these findings, we have demonstrated that LSG mucins from patients with SjD show decreased levels of sulfated oligosaccharides (21, 22). We associated these results with decreased enzymatic activity of Gal3-O-sulfotransferases (Gal3ST) of the Golgi apparatus, despite their normal transcript and protein levels in LSGs from patients with SjD (23).

Altered mucin O-glycosylation is reported in autoimmune diseases (21, 24) and cancers (25, 26). For example, elevated sialyl-T oligosaccharide and decreased core 2 oligosaccharide levels have been observed in prostate adenocarcinoma, with the degree of abnormality varying according to the histological grade of the tumor (26). Both types of oligosaccharides are synthesized from the core 1 structure. If C2GnT in the initial compartments of the Golgi apparatus acts first, the core 2 structure is synthesized. If the sialyltransferase ST3GalT in the terminal compartments of the Golgi apparatus acts first, sialyl-T is synthesized. Thus, the increase in sialyl-T oligosaccharides occurs in association with C2GnT enzyme deficiency (27). In prostate cancer, cell vesicles carrying C2GnT are exclusively recruited by Giantin to the Golgi apparatus, whereas vesicles carrying the ST3GalT enzyme can be recruited by Giantin or GM130 (26). Abnormal C2GnT expression levels have not been observed in advanced prostate adenocarcinoma cells; however, this enzyme is not localized in the Golgi apparatus, which may explain the decreased levels of core 2 oligosaccharides in these cells (26).

Giantin is a dimeric protein with a large cytosolic domain, a transmembrane domain, and a C-terminal disulfide-linked luminal domain (28). In tumor cells with abnormal C2GnT localization, Giantin is present in its monomeric form and does not form dimers, affecting C2GnT function and preventing its appropriate destination within the Golgi apparatus (26). It remains unknown whether Giantin directs the localization of other Golgi enzymes. Here, we showed the abnormal localization of Giantin in LSGs from patients with SjD. Coimmunoprecipitation assays showed protein associations between Giantin and Gal3STs. In Giantin-knockout (Giantin-KO) and -knockdown cells, we observed that Gal3ST enzymes changed their localization, suggesting that Giantin could participate in the tethering of vesicles transporting Gal3ST enzymes to the Golgi apparatus. Although levels of Giantin do not decrease, its altered localization could affect the correct tethering of the vesicles transporting Gal3STs to the Golgi apparatus, which could explain the decreased enzymatic activity of Gal3ST and the decreased sulfation of MUC5B observed in LSGs from patients with SjD.

## Results

### Altered distribution of Giantin in LSGs of patients with SjD.

**Figure 1:**
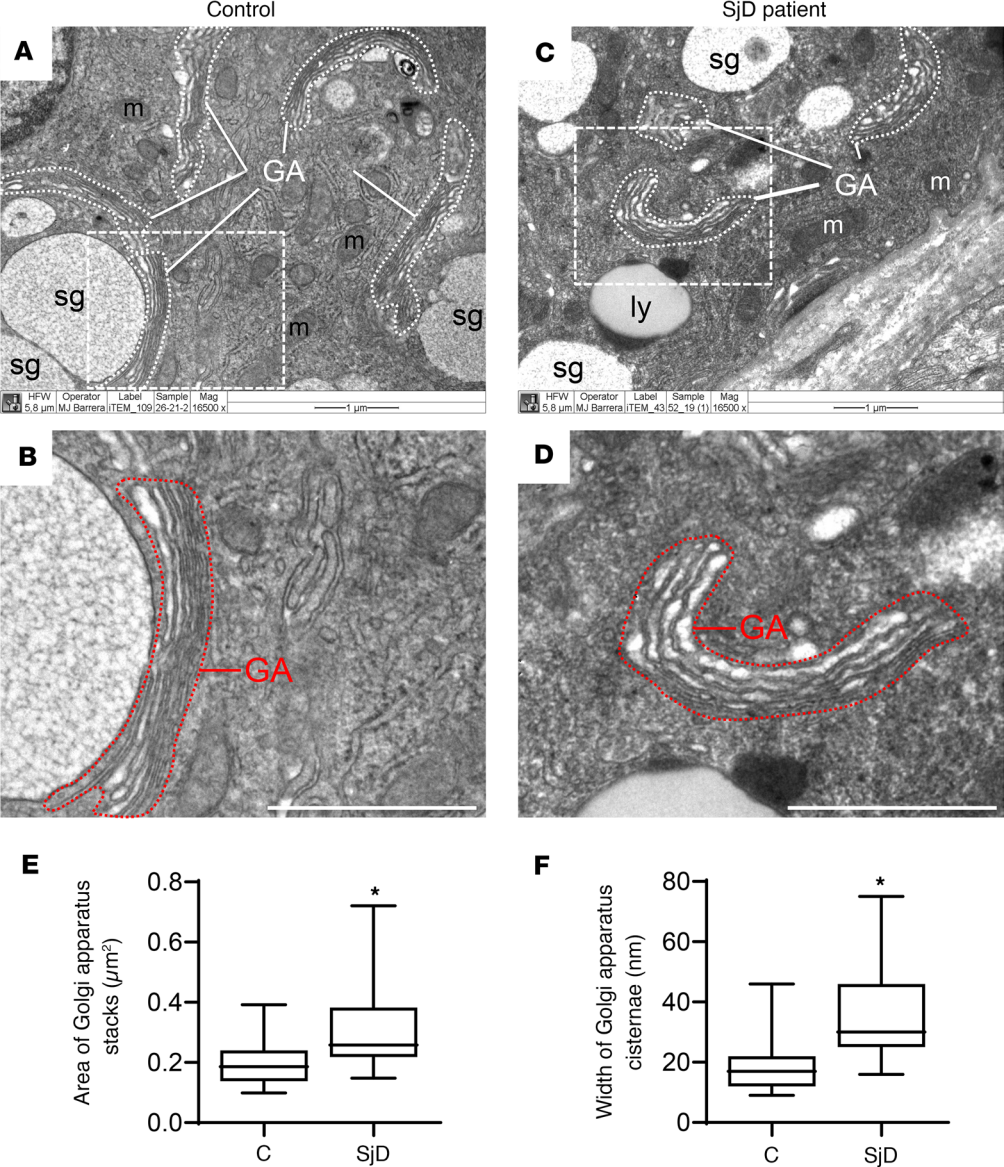
Swelling of the Golgi apparatus cisterns in epithelial cells of LSGs from patients with SjD. (**A**–**D**) Representative transmission electron micrographs showing the Golgi apparatus (GA) stacks surrounded by white dashed lines in salivary epithelial cells of an individual acting as a control and a patient with SjD. Scale bars: 1 μm. m, mitochondria; sg, secretory granule; ly, lysosome. (**B** and **D**) Higher-magnification images of regions bound by dashed lines in **A** and **C**, showing the GA stacks bound by red dashed lines. (**E** and **F**) Box plots showing the area of the Golgi apparatus stacks and the width of the Golgi apparatus cisternae. The quantification was performed on ultrathin sections from 3 individuals acting as controls and 6 patients with SjD. Boxes represent the 25th–75th percentiles; the lines within the boxes represent the median; and the whiskers represent the minimum and maximum. *P < 0.05 was considered significant using the Mann-Whitney test.

**Figure 2:**
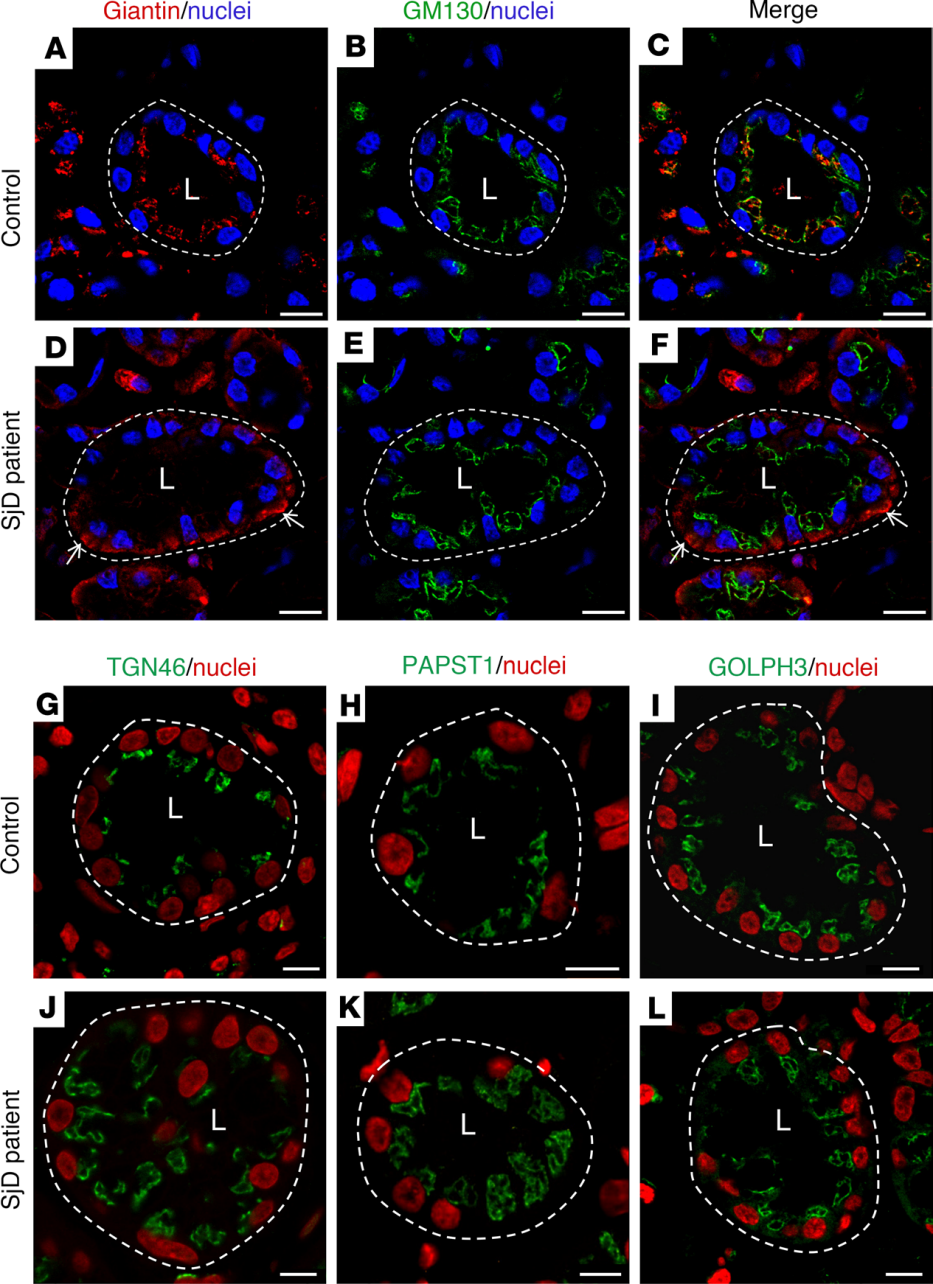
Altered localization of Giantin in LSGs from patients with SjD. (**A**–**F**) Representative micrographs of double staining of Giantin (red) and GM130 (green) in a section of LSG from an individual acting as a control (**A**–**C**) and a patient with SjD (**D**–**F**). White arrows show Giantin staining in the basolateral region. (**G**–**L**) Representative images of TGN46, PAPST1, and GOLPH3 in LSG sections from individuals acting as controls (**G**–**I**) and patients with SjD (**J**–**L**). Hoechst 33342 was used for nuclear staining. The white dashed lines indicate acinar boundaries. L, lumen. Scale bars: 20 μm (**A**–**F**); 10 μm (**G**–**L**).

**Figure 3:**
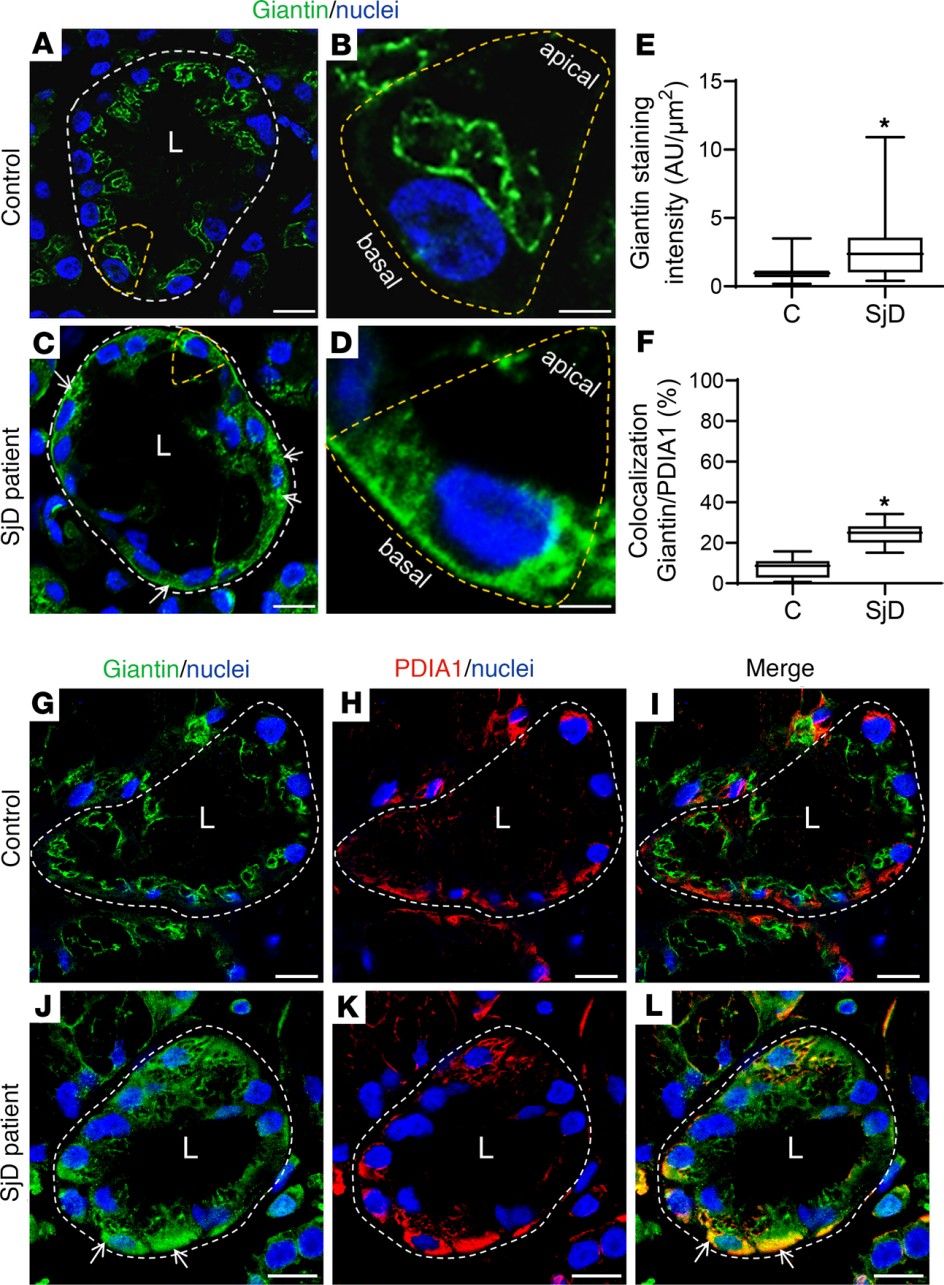
Increased Giantin protein levels in LSGs from patients with SjD. (**A–D**) Representative images of Giantin (green) staining in LSG sections from individuals acting as controls and patients with SjD. (**B** and **D**) Higher-magnification images of epithelial cells surrounded by yellow dashed lines in **A** and **C**, respectively. (**E**) Giantin staining was quantified in acini from LSG sections of 5 individuals acting as controls and 5 patients with SjD. (**F**) Colocalization analysis of Giantin and PDIA1 in acini from LSG sections of 5 individuals acting as controls and 5 patients with SjD. Boxes represent the 25th–75th percentiles; the lines within the boxes represent the median; and the whiskers represent the minimum and maximum. *P < 0.05 was considered significant using the Mann-Whitney test. (**G**–**L**) Representative micrographs of double staining of Giantin (green) and PDIA1 (red) in a section of LSG from individuals acting as controls (**G**–**I**) and patients with SjD (**J**–**L**). Hoechst 33342 (blue) was used for nuclear staining. White arrows show Giantin staining in the basolateral region. The broken lines indicate acinar boundaries. L, lumen. Scale bars: 20 μm (**A** and **C**); 5 μm (**B** and **D**); 10 μm (**G**–**I** and **J**–**L**).

**Figure 4:**
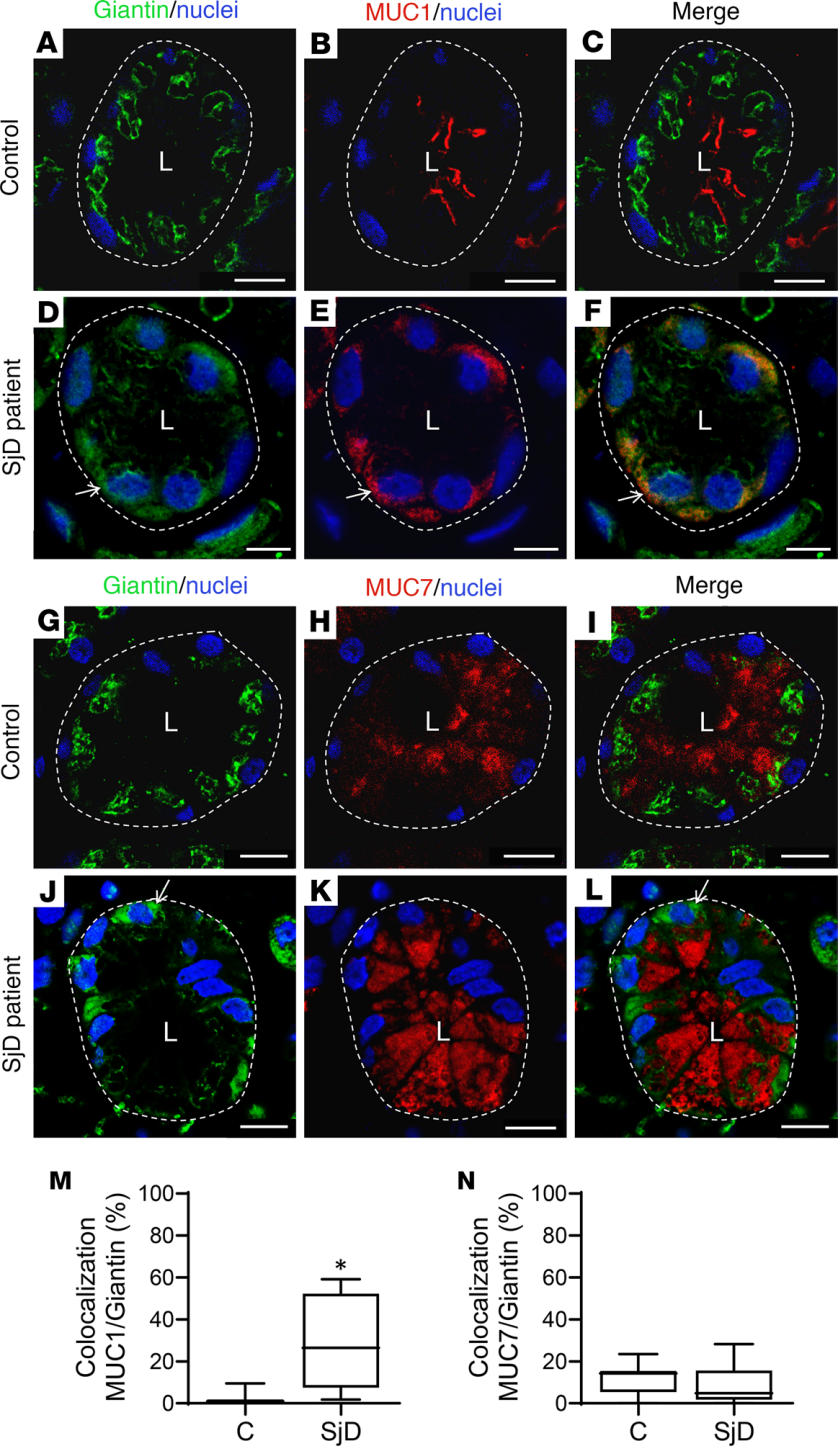
Altered localization of Giantin and mucins in LSGs from patients with SjD. (**A**–**F**) Representative micrographs of double staining of Giantin (green) and MUC1 (red) in a section of LSG from an individual acting as a control (**A**–**C**) and a patient with SjD (**D**–**F**). (**G**–**I**) Representative micrographs of double staining of Giantin (green) and MUC7 (red) in a section of LSG from an individual acting as a control (**G**–**I**) and patient with SjD (**J**–**I**). Hoechst 33342 (blue) was used for nuclear staining. The dashed lines indicate acinar boundaries. L, lumen. Scale bars: 10 μm. (**M** and **N**) Colocalization analysis of Giantin and mucins in acini from LSG sections of 5 individuals acting as controls and 5 patients with SjD. Boxes represent the 25th–75th percentiles; the lines within the boxes represent the median; and the whiskers represent the minimum and maximum. *P < 0.05 was considered significant using the Mann-Whitney test.

Using transmission electron microscopy, we observed a swelling of the Golgi apparatus cisterns in epithelial cells of LSGs from patients with SjD when compared with individuals acting as controls (Figure 1). In polarized epithelial cells, such as the acinar and ductal cells from LSG, the ER is mainly located in the basolateral region, while the Golgi apparatus is located in the supranuclear region. As expected, in LSGs from all the individuals acting as controls, the Golgi markers Giantin and GM130 were observed in the supranuclear region (Figure 2, A–C, and Figure 3, A and B), while in LSGs from some patients with SjD, Giantin staining mainly appeared in the basolateral region (Figure 2, D–F, and Figure 3, C and D), colocalizing with the ER marker PDIA1 (Figure 3, F–L). Additionally, quantification of Giantin staining showed a significant increase in LSGs from patients with SjD (*P* < 0.0001) (Figure 3, A–E). In the LSGs from patients with SjD with mislocalized Giantin, we did not observe changes in the localization of TGN46, PAPST1, or GOLPH3 (Figure 2, G–L). TGN46 is a type I protein involved in post-TGN vesicle formation (29). PAPST1 is a specific transporter of 3′-phosphoadenosine 5′-phosphosulfate (PAPS), a universal sulfuryl donor for sulfation (30). GOLPH3 is a phosphoprotein that functions in secretory trafficking at the Golgi (31). The altered localization of Giantin observed in some LSGs from patients with SjD was confirmed by double staining with the salivary mucins MUC1 and MUC7 (Figure 4). We have previously found that MUC1 is overexpressed and accumulates in the ER of LSGs from patients with SjD (20), while MUC7 is accumulated in the cytoplasm (32), and ectopically secreted into the extracellular matrix (33).

### Giantin forms protein associations with Gal3ST enzymes.

**Figure 5:**
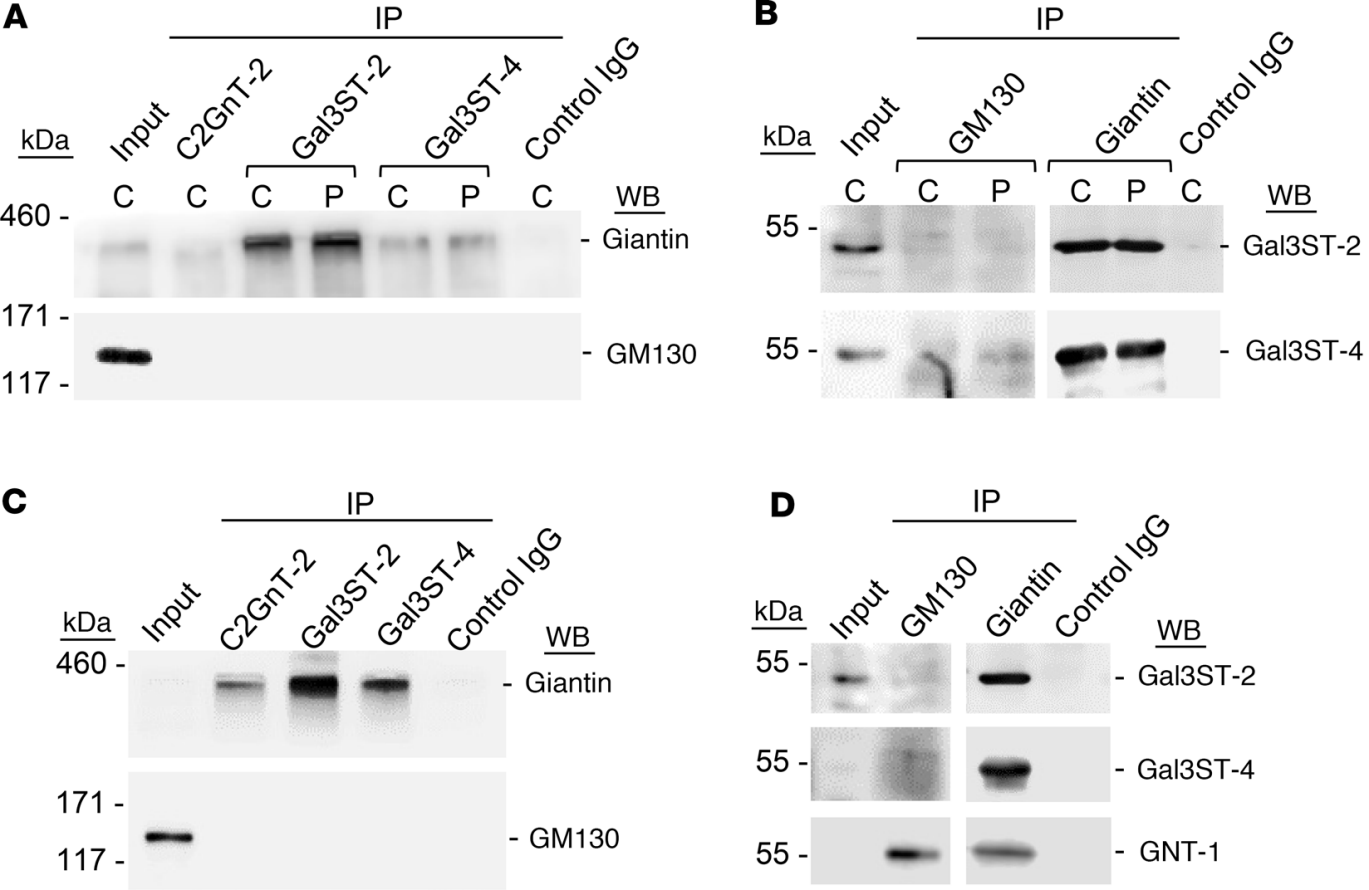
Formation of protein associations between Giantin and Gal3STs. (**A**) C2GNT-2, Gal3ST-2, and Gal3ST-4 were immunoprecipitated (IP) from protein extracts of LSG from individuals acting as controls (C) and patients with SjD (P) and then analyzed by Western blot (WB) with anti-Giantin (~376 kDa) or anti-GM130 (130 kDa) antibodies. (**B**) GM130 and Giantin were IP from protein extracts of LSGs from individuals acting as controls and patients with SjD (P) and then analyzed by WB with anti–Gal3ST-2 (46 kDa) and anti–Gal3ST-4 (54 kDa) antibodies. (**C**) C2GNT-2, Gal3ST-2, and Gal3ST-4 were IP from protein extracts of HSG cells and then analyzed by WB with anti-Giantin or anti-GM130 antibodies. (**D**) GM130 and Giantin were IP from protein extracts of HSG cells and then analyzed by WB with Gal3ST-2 and Gal3ST-4 antibodies. The enzyme GNT-1 (57 kDa) was used as a positive control of IP GM130 and Giantin. For all experiments, the control IgG was α6 integrin.

Upon immunoprecipitation (IP), Giantin was detected with Gal3ST-2 or Gal3ST-4 antibodies, whereas GM130 was not detected in protein extracts from LSG (Figure 5A). IP with C2GnT-2 antibodies was used as a positive control to detect Giantin, in agreement with a previous report (17). Likewise, when IP was performed with anti-Giantin antibodies, intense bands were observed for Gal3ST2 and Gal3ST4, whereas when IP was performed with anti-GM130 antibodies, there were no Gal3ST-2 bands and very weak Gal3ST-4 bands (Figure 5B). To determine if protein associations between Giantin and Gal3STs observed in LSGs from patients with SjD and individuals acting as controls also occurred in HSG cells, co-IP was performed using protein extracts from HSG cells. The results showed a band corresponding to Giantin upon IP with Gal3ST-2 or Gal3ST-4 antibodies, whereas GM130 was not detected (Figure 5C). IP with C2GnT-2 antibodies was used as a positive control (17). Likewise, when IP was performed with anti-Giantin antibodies, intense bands were observed when detecting Gal3ST2 and Gal3ST4, whereas when IP was performed with anti-GM130 antibodies, there were no Gal3ST-2 bands and very weak Gal3ST-4 bands (Figure 5D). GNT-1 enzyme, which was detected upon IP of GM130 and Giantin in HSG cells, was used as a positive control. These results suggest that Giantin forms protein associations with Gal3STs and may be involved in their correct localization.

### Giantin mediates the Golgi localization of Gal3STs.

**Figure 6:**
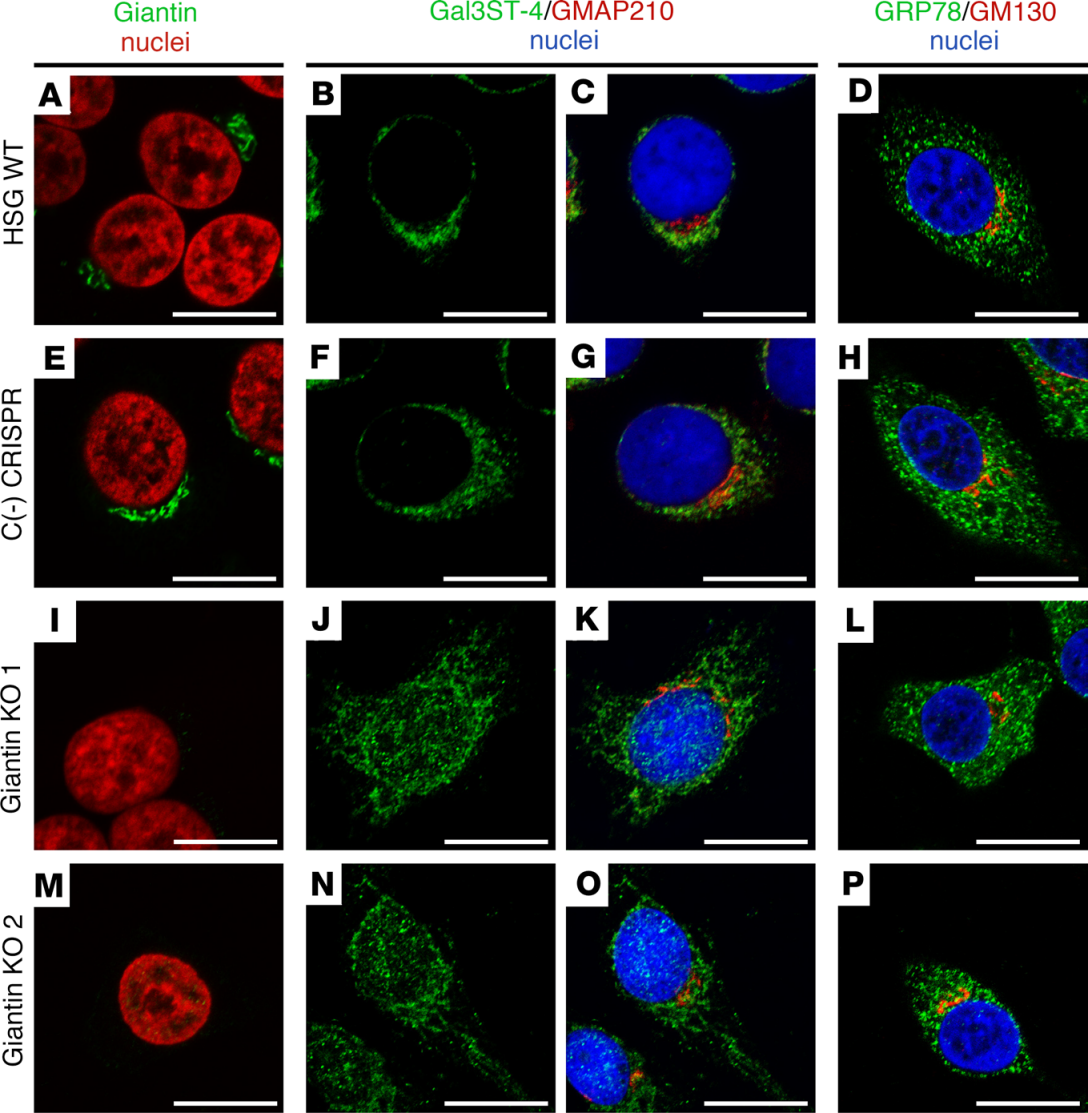
Altered Gal3ST-4 localization in Giantin-KO cells. (**A**–**D**) Representative micrographs of WT HSG cells, (**E**–**H**) negative control [C(-)] CRISPR/Cas9 HSG cells, and (**I**–**P**) CRISPR/Cas9 Giantin-KO cells. **A**, **E**, **I**, and **M** show Giantin (green) and nuclei (red). **B**, **F**, **J**, and **N** show the green channel of the double staining of Gal3ST-4 (green) and GMAP210 (red). **C**, **G**, **K**, and **O** show the merged RGB channels. **D**, **H**, **L**, and **P** show the merged RGB channels of the double staining of GRP78 (green) and GM130 (red). Hoechst 33342 (blue) was used for nuclear staining. Scale bars: 10 μm.

As Giantin has an atypical localization in LSGs from patients with SjD, we silenced Giantin with two different experimental strategies to mimic Giantin malfunction in vitro. Using CRISPR/CAS9, we established 2 different Giantin-KO cell lines (Supplemental Figure 1; supplemental material available online with this article; https://doi.org/10.1172/jci.insight.171585DS1), and using shRNAs we established a Giantin-knockdown (Giantin-KD) cell line (sh-Giantin) and a GM130 KD cell line (sh-GM130) (Supplemental Figure 2). To evaluate whether Giantin silencing affected the localization of Gal3ST-2 or Gal3ST-4, these enzymes were detected by immunofluorescence in WT HSG cells, Giantin-KO cells, and Giantin- or GM130-KD cells. In WT and CRISPR-negative control cells, Gal3ST-4 was mainly localized adjacent to the nucleus, showing the same polarity as the GMAP210 golgin (Figure 6, A–H, and Supplemental Figure 3). In Giantin-KO cells, Gal3ST-4 was observed throughout the nuclei and the cytoplasm (Figure 6, I–P, and Supplemental Figure 4). The cytoplasmic Gal3ST-4 staining in Giantin-KO cells was quite similar to the localization of the ER marker GRP78 (Figure 6, J–P). We were not able to perform double staining of Gal3ST-4 and ER markers because different fixation protocols are necessary for each immunostaining.

**Figure 7:**
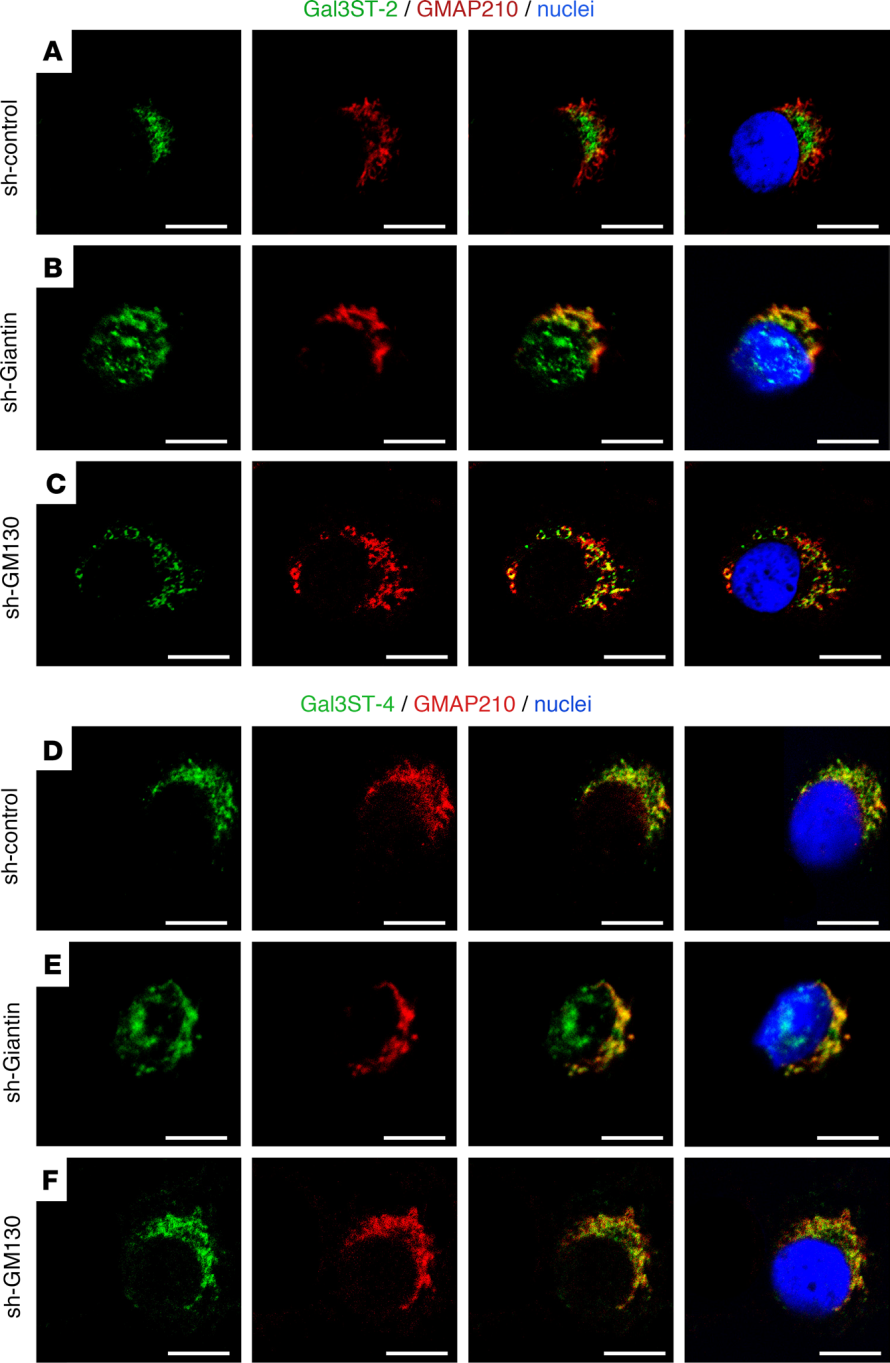
Altered Gal3ST-2 and Gal3ST-4 localization in sh-Giantin cells. Representative micrographs of double staining of Gal3STs (green) and GMAP210 (red) in HSG cells. (**A**) In sh-control cells, Gal3ST-2 is located adjacent to the nucleus and partially colocalizes with GMAP210. (**B**) In sh-Giantin cells, Gal3ST-2 distribution changes, with diffuse staining “on the nucleus.” (**C**) Gal3ST-2 detection in sh-GM130 cells shows a distribution similar to sh-control cells. (**D**) In sh-control cells, Gal3ST-4 is located adjacent to the nucleus and partially colocalizes with GMAP210. (**E**) In sh-Giantin cells, Gal3ST-4 distribution changes, with diffuse staining “on the nucleus.” (**F**) Gal3ST-4 detection in sh-GM130 cells shows a distribution similar to that of sh-control cells. Hoechst 33342 (blue) was used for nuclear staining. Scale bars: 10 μm.

In most sh-control cells, Gal3ST-2 localized adjacent to the nucleus in a “crescent moon shape” (Figure 7A), but in a smaller percentage of cells, it localized adjacent to the nucleus in a ring shape (Supplemental Figure 5). The localization of Gal3ST-2 in sh-GM130 cells was quite similar to that of sh-control cells (Figure 7C). However, sh-Giantin cells apparently showed staining “on the nucleus” (Figure 7B). We preferred to use this “on the nucleus” description instead of “in the nucleus,” because in the Z-stacks series of images we did not see overlapping of the Gal3STs and Hoechst staining (see Supplemental Video 1). The results observed for Gal3ST-4 were like those of Gal3ST-2 (Figure 7, D–F, and Supplemental Figure 6), suggesting that the silencing of Giantin and not GM130 affects the correct localization of these enzymes in the Golgi apparatus.

### Localization of Gal3STs is also affected by Golgi stress.

**Figure 8:**
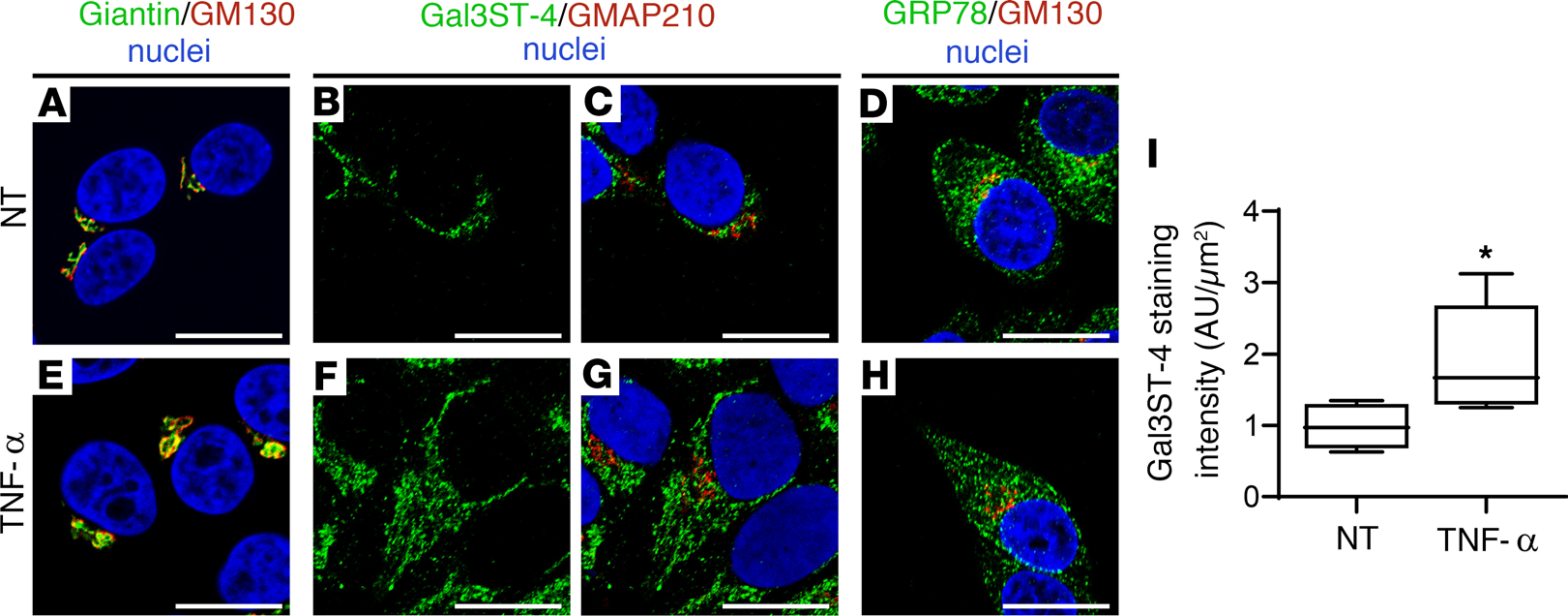
Altered Gal3ST-4 localization in TNF-α–stimulated HSG cells. Representative micrographs of (**A**–**D**) nontreated (NT) HSG cells or (**E**–**H**) HSG cells stimulated with 10 ng/mL TNF-α for 24 hours. (**A** and **E**) The merged RGB channels of the double staining of Giantin (green) and GM130 (red). (**B** and **F**) The green channel of the double staining of Gal3ST-4 (green) and GMAP210 (red). (**C** and **G**) The merged RGB channels. (**D** and **H**) The merged RGB channels of the double staining of GRP78 (green) and GM130 (red). Hoechst 33342 (blue) was used for nuclear staining. Scale bars: 10 μm. (**I**) Box plot showing the quantification of Gal3ST-4 staining. Boxes represent the 25th–75th percentiles; the lines within the boxes represent the median; and the whiskers represent the minimum and maximum. *P < 0.05 was considered significant using the Mann-Whitney test.

Increasing evidence has suggested that the secretion of large amounts of proteins overwhelms the glycosylation and vesicular transport machinery of the Golgi apparatus, leading to a Golgi stress response. This stress response increases the expression of Golgi-resident proteins, which helps to restore Golgi homeostasis (34). LSGs from patients with SjD show overexpression of mucins, which has been replicated in vitro under TNF-α stimulation (20). TNF-α is one of the main proinflammatory cytokines overexpressed in salivary glands, saliva, and serum from patients with SjD (35–37). In TNF-α–stimulated cells, we observed a perinuclear and cytoplasmic localization of Gal3ST-4, quite like the localization observed for GRP78 (Figure 8, A–H). In addition, an increased staining intensity of Gal3ST-4 was observed in TNF-α–stimulated cells (Figure 8I). Currently, the known signaling pathways of the Golgi stress response include TFE3, mucin, proteoglycan, HSP47, CREB3, and PERK (38–45). TFE3 is a basic helix-loop-helix transcription factor that upon Golgi stress is translocated to the nucleus, where it binds to a cis-acting enhancer element called the Golgi apparatus stress response element (GASE) (38). GASE (consensus sequence ACGTGGC) is present in the promoters of several Golgi-resident proteins, such as Giantin and GM130 (39, 40). Here, we observed increased mRNA levels of TFE3 and Giantin in TNF-α–stimulated cells (Supplemental Figure 7), suggesting that TNF-α is activating the Golgi stress response in vitro and may be acting as a Golgi stress agent in LSGs from patients with SjD.

### Decreased sulfated glycans of MUC5B in LSGs of patients with SjD.

**Figure 9:**
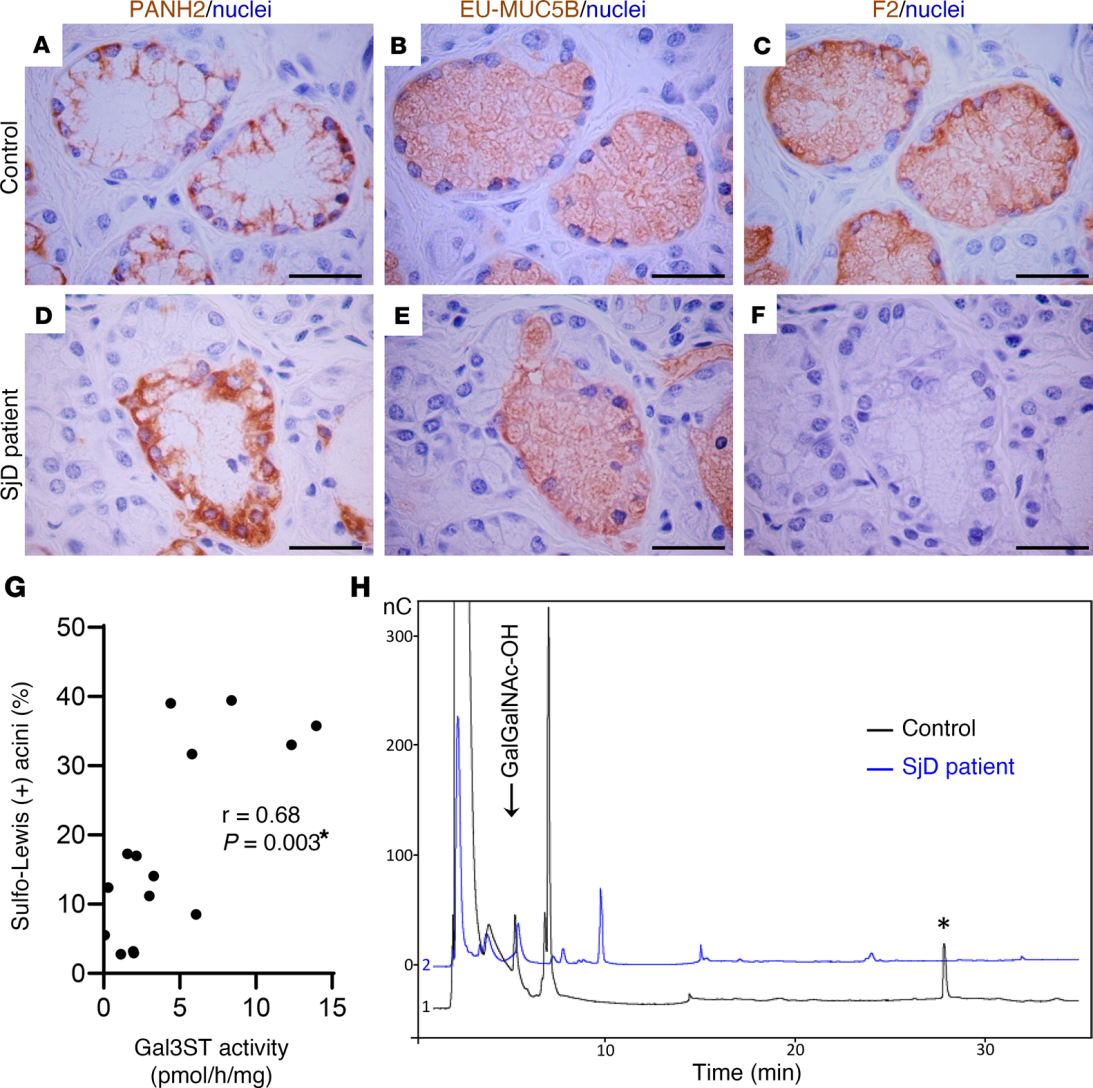
Decreased MUC5B sulfated O-glycans in LSGs from patients with SjD. (**A**–**F**) Representative images of immunohistochemical detection of partially glycosylated MUC5B (PANH2) (**A** and **D**), the MUC5B polypeptide backbone independent of its glycosylation status (EU-MUC5Bb) (**B** and **E**), and sulfo-Lewis a and c residues (F2) (**C** and **F**) in serial sections of LSGs from individuals acting as controls (**A**–**C**) and patients with SjD (**D**–**F**). Scale bars: 50 μm. (**G**) Spearman’s correlation between Gal3STs activity and the percentage of sulfo-Lewis a and c (F2)-positive acini. *P < 0.05 was considered significant. (**H**) Oligosaccharide mixtures of MUC5B obtained by reductive β-elimination analyzed by HPAEC-PAD in LSGs from individuals acting as controls (black line) and patients with SjD (blue line). The asterisk in **H** shows a peak detected in control samples but not in SjD samples. nC, chromatographic fingerprint of the signal.

Antibodies directed against different peptide and glycosidic epitopes of MUC5B were used (Supplemental Table 4) in serial sections of LSGs from 6 patients with SjD and 9 individuals acting as controls. PANH2 antibody recognizes an epitope located in the peptide backbone of the highly glycosylated region of MUC5B and thus only reacts when this region is partially glycosylated. The EU-MUC5Bb antibody recognizes the RNREQVGKFKMC sequence of the CYS domains of MUC5B, detecting the polypeptide backbone independent of its glycosylation status. The F2 antibody recognizes the SO_3_Gal1-3GlcNAc epitope of Sulfo-Lewis^a^ (SO_3_Galβ1-3[Fucα1-4]GlcNAc) and Sulfo-Lewis^c^ (SO_3_Galβ1-3GlcNAc) oligosaccharides. As previously reported in LSGs from individuals acting as controls (21), PANH2 staining was observed in the basal region of mucous acinar cells, where the endoplasmic reticulum is located and apomucin synthesis occurs (Figure 9A and Supplemental Figure 8). EU-MUC5Bb and F2 staining showed a distribution throughout the cytoplasm of mucous acinar cells, as they continued recognizing mucin epitopes during its transit through the Golgi apparatus (Figure 9, B and C, and Supplemental Figure 8). In LSGs from patients with SjD, stronger PANH2 staining throughout the cytoplasm implies that the antibody still has access to the apomucin even after it has transited through the Golgi apparatus, suggesting decreased glycosylation of MUC5B in these cells (Figure 9D and Supplemental Figure 8). The total number of mucosal acini (PANH2 and EU-MUC5Bb positive) and the number of Sulfo-Lewis–positive (F2-positive) acini were quantified by two independent observers. We found a significant decrease in the percentage of F2-positive mucous acini in LSGs from patients with SjD (9.7% ± 6.7%), compared with those from individuals acting as controls (24% ± 14%) (*P* = 0.044) (Figure 9, D–F, and Supplemental Figure 8). This analysis was performed using the same sample we previously used to determine Gal3ST enzymatic activity (23) (Supplemental Figure 8). Moreover, there was a positive correlation between Gal3ST activity and the percentage of positive Sulfo-Lewis–positive acini (Figure 9G).

**Figure 10:**
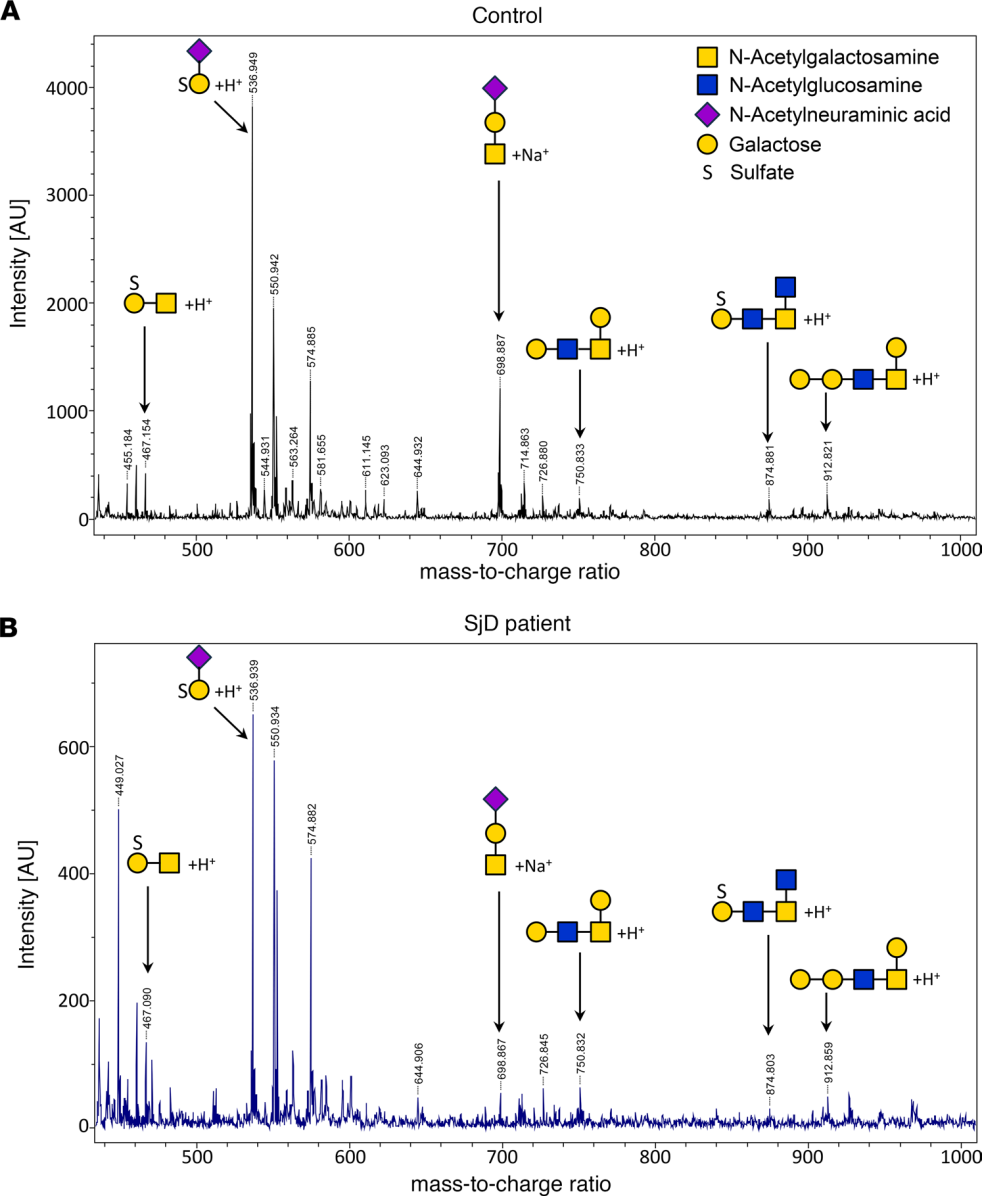
Decreased abundance of MUC5B-associated oligosaccharides in LSGs from patients with SjD. The oligosaccharide mixtures of MUC5B, obtained by reductive β-elimination in LSGs from individuals acting as controls (**A**) and patients with SjD (**B**), were also analyzed by UV-MALDI-TOF mass spectrometry. Signals for sulfated and nonsulfated oligosaccharides were detected; however, the abundance observed in samples from patients with SjD showed a substantial reduction (spectra are shown on different scales). O-glycan structures are depicted using the symbol nomenclature for glycans (SNFG).

MUC5B from LSGs from patients with SjD and individuals acting as controls were analyzed by SDS-PAGE and transferred to PVDF membranes. The band corresponding to this high-molecular-weight protein was excised and subjected to reductive β-elimination to release the O-glycan structures. The oligosaccharide mixtures obtained were analyzed by HPAEC-PAD, revealing different profiles of the samples (Figure 9H). In control samples, a significant peak was observed at longer retention times (marked with an asterisk), which was not observed in samples from patients with SjD. In the latter, a higher signal appeared at a retention time near 10 minutes, which was absent in the control samples (Figure 9H). These results indicate the presence of larger or more highly charged oligosaccharides (sulfated and/or sialylated) in samples from individuals acting as controls. The oligosaccharides were also analyzed by UV-MALDI-TOF mass spectrometry, which allowed us to identify the more abundant species. Both sulfate-containing and nonsulfate oligosaccharides were found in samples from individuals acting as controls and patients with SjD. The relative abundance of oligosaccharides showed smaller peaks for each structure in LSGs from patients with SjD. Therefore, we adjusted the spectra to different scales to discern these small peaks (Figure 10). These results confirm the decreased glycosylation and sulfation of MUC5B in LSGs from patients with SjD.

## Discussion

Numerous glycosyltransferases, glycosidases, and sugar nucleotide transporters involved in the synthesis of mucin-associated oligosaccharides reside in the Golgi apparatus. Glycosyltransferases must act on an acceptor substrate at the right time and in the right place, so the different enzymes are compartmentalized in different membranes of the Golgi apparatus (13). Each compartment is surrounded by numerous vesicles that perform the retrograde transport of enzymes from the Golgi apparatus back to their compartment of residence. In the secretory pathway, vesicles are recruited to the target membrane to ensure specificity and fusion efficiency. The factors that recruit vesicles are known as tethering factors and act in concert with specific small RabGTPases to bring vesicles closer to the acceptor membrane, promoting the formation of SNARE complexes and facilitating membrane fusion. In the Golgi apparatus, there are two main types of tethering factors. One of them, the golgins, use their extensive coiled-coil helices to capture vesicles and are essentially involved in the anterograde trafficking of proteins (46). The other factor is a group of proteins known as multisubunit tethering complexes (MTC), which includes the conserved oligomeric Golgi (COG) complex and is key in retrograde trafficking and enzyme retrieval from the Golgi apparatus (15).

Giantin belongs to the golgin family of proteins, which are localized on the cytosolic face of the Golgi apparatus membrane (46). Interestingly, the presence of autoantibodies directed against several golgins has been described in the sera of some patients with SjD (47). These autoantibodies mainly recognize Giantin, and a smaller percentage of them also target GM130, Golgin-245, Golgin-160, and Golgin-97 (48, 49). The mechanism by which these golgins become self-antigens in autoimmune diseases is unclear. One possible explanation is that these proteins are recognized by immune system cells in apoptotic or necrotic cellular debris. Unlike other self-antigens frequently found in patients with SjD, such as Ro60/SSA, golgins have not been observed in apoptotic blebs (50, 51). In SjD, the generation of other autoantibodies, such as anti-fodrin and anti-muscarinic M3 receptor, has been associated with the appearance of proteolytic fragments of these autoantigens during cell death (52). In this context, immunogenic proteolytic fragments of Giantin have been observed in Jurkat T cells stimulated with the cell death inducer Staurosporine, an effect that disappears in the presence of caspase inhibitors (50).

Here, Giantin was observed in the supranuclear region of LSG epithelial cells from individuals acting as controls, whereas in some patients with SjD it was additionally detected in the perinuclear basolateral region, colocalizing with the ER marker PDIA1. No changes in Giantin localization were observed in LSGs from 5 patients with sarcoidosis, suggesting that this alteration is not a generalized phenomenon occurring in other inflammatory diseases involving salivary glands (Supplemental Figure 9 and Supplemental Table 1). The partial retention of Giantin in the ER would affect its vesicle tethering mechanism, which transports some enzymes of the Golgi apparatus. At present, the golgin involved in the correct localization of sulfotransferases (Gal3ST) is unknown. LSGs from patients with SjD have shown a significant decrease in Gal3ST activity without changes in their transcript or protein levels with respect to controls (23). Unfortunately, we have not been able to detect Gal3STs by immunofluorescence in LSG biopsies from patients with SjD owing to a lack of appropriate antibodies. One possible hypothesis is that retention of Giantin in the ER could prevent Gal3STs from reaching their correct localization in the Golgi apparatus. The results of this study showed that Giantin coimmunoprecipitates with Gal3ST-2 and Gal3ST-4, and this is the first evidence to our knowledge suggesting that Giantin could be involved in the tethering of vesicles transporting these enzymes to the Golgi apparatus. In addition, Giantin silencing produced an alteration in the localization of Gal3ST-2 and Gal3ST-4 enzymes in salivary gland epithelial cells, whereas it did not alter the localization of the cis-Golgi marker, GMAP210. The localization of Gal3STs was not altered when GM130 was silenced. These results suggest that Giantin may participate in the tethering of vesicles transporting Gal3ST-2 and Gal3ST-4 to the Golgi apparatus in salivary gland epithelial cells.

Giantin is a dimeric protein with a large cytosolic domain (>350 kDa) and a C-terminal transmembrane domain, where disulfide bond formation occurs (28). During its synthesis, it is anchored to the ER membrane and then transported to the Golgi apparatus (53). GM130 is a peripheral protein that associates with cis-Golgi cisternae through interaction with GRASP65. Giantin and GM130 interact with p115 to enable vesicular transport from the ER to the ERGIC or to the Golgi apparatus and between Golgi apparatus cisternae (54). The inhibition of transport from the ER to the Golgi apparatus by a dominant-negative Sar1 induces partial redistribution of Giantin to the ER, while GM130, GRASP65, and TGN46 remain localized in punctate structures that would correspond to remnants of the Golgi apparatus (55). This accumulation of Giantin in the ER, evidenced by immunofluorescence and subcellular fractionation, is not caused by the presence of newly synthesized proteins, since this effect also occurs in the presence of cycloheximide (55). In LSGs from patients with SjD we observed that Giantin was localized in the ER, whereas GM130 and TGN46 showed a well-defined staining in the supranuclear region of acinar cells, suggesting that alterations in the trafficking machinery from the ER to the Golgi apparatus could explain the differential behavior of these proteins.

Unexpectedly, GM130 transcript levels were significantly increased in sh-Giantin cells (Supplemental Figure 7). Giantin silencing could generate a deficit in Golgi apparatus function, which then would activate the Golgi stress response, accounting for the increased GM130 transcript levels. The Golgi stress response is a homeostatic mechanism by which cells increase the capacity of the Golgi apparatus, inducing the transcription of genes that are relevant for Golgi structure and function, such as glycosyltransferases, structural proteins, and vesicular transport components (40). Here, we determined the transcript levels of Golgi stress markers, such as the transcription factors TFE3 and CREB3 and the Golgi-resident proteins GCP60 and p115. All of these transcript levels were significantly increased in sh-Giantin cells (Supplemental Figure 7), suggesting that the Golgi stress response is active upon Giantin silencing. We also analyzed the levels of glycosyltransferases (target genes of the Golgi stress response) in LSGs from patients with SjD and individuals acting as controls and found a significant increase of GCNT1 transcript levels in LSGs from patients with SjD (Supplemental Figure 7). We have previously reported increased MUC1 mRNA and protein levels in LSGs from patients with SjD, together with an accumulation of MUC1 in the ER, colocalizing and coimmunoprecipitating with GRP78 (20). The incubation of 3D acini with TNF-α or IFN-γ increases the expression and ER accumulation of MUC1 (20), and here we show increased Giantin mRNA levels upon TNF-α stimulation. This proinflammatory stimulus also changed the localization and staining intensity of Gal3ST-4, suggesting that TNF-α may be acting as a Golgi stress agent in LSGs from patients with SjD. Interestingly, by transmission electron microscopy, we observed a swelling of the Golgi apparatus cisterns in epithelial cells of LSGs from patients with SjD, suggesting that LSGs from patients with SjD have a stress condition that involves both the ER and the Golgi apparatus.

Sulfation of mucins such as MUC5B is essential for lubrication and water retention in the oral mucosa. MUC5B sulfation is catalyzed by Gal3STs, which shows decreased activity in LSGs from patients with SjD (23). Here, we showed a correlation between Gal3ST activity levels and the number of Sulfo-Lewis–positive acini in LSG. In addition, mass spectrometry analysis showed decreased levels of oligosaccharides associated with MUC5B, confirming the decreased glycosylation and sulfation of this mucin in LSGs from patients with SjD. Altered localization of Gal3STs could affect their activity due to the alteration of the optimal microenvironment for their function. Our results suggest that Giantin could act as a tethering factor of the vesicles that transport these enzymes to the Golgi apparatus, and its mislocalization could affect the sulfation of salivary mucins in LSGs from patients with SjD. MUC5B is a major gel-forming mucin present in saliva, contributing to its viscoelastic and lubricating properties. The changes reported here may assist in understanding the oral signs and symptoms reported by patients with SjD.

## Methods

### Sex as a biological variable.

The groups studied included more female participants. This is because SjD is about 10 times more common in women than in men (1).

### Patients with SjD and participants acting as controls.

The study group included 24 patients diagnosed with SjD, according to the 2016 ACR/EULAR Classification Criteria (56). The control group involved 24 individuals who did not fulfill the SjD classification criteria, without systemic diseases, and whose lip biopsy analysis was normal or revealed mild diffuse chronic sialadenitis. A detailed description of demographic, serological, and histological characteristics of the patients with SjD and individuals acting as controls is summarized in Supplemental Table 1.

### Biopsies.

The LSG biopsies were performed according to Daniels (57). Following surgery, samples were immediately frozen in liquid nitrogen or fixed for morphological and immunofluorescence studies.

### Cell culture.

Human submandibular gland (HSG) cells were provided by Bruce Baum (National Institute of Dental and Craniofacial Research, NIH, Bethesda, MD, USA) and cultured as a monolayer, as previously described (58). These cells were incubated with or without 10 ng/mL human recombinant TNF-α (Biolegend), in serum-free medium for 24 hours and then lysed for RNA isolation or fixed for immunofluorescence studies. The GOLB1-KO cell line was generated using CRISPR/Cas9. A sgRNA targeting exon 8 of the GOLGB1 gene (encoding Giantin protein) was designed using the online designing tools CHOP-CHOP (http://chopchop.cbu.uib.no) (59) and CRISPOR (http://crispor.tefor.net/) (60) (Supplemental Table 2). A nontargeting gRNA was selected from the literature to serve as a CRISPR-negative control (61) (Supplemental Table 2). The sgRNA sequences were annealed and cloned into the eSpCas9-T2A-Puro plasmid (Addgene) using T4 DNA ligase (New England BioLabs). HSG cells were transfected with 0.5 μg sgRNA-eSpCas9 using Viafect (Promega). After 24 hours, cells were selected with 1 μg/mL puromycin for 3 days and then the colonies were isolated by limiting dilution in a 96-well plate. After 7–14 days, the plate was scanned to verify the presence of single-cell (monoclonal) colonies. The colonies were then expanded, and Giantin KO was verified by Sanger sequencing, Western blot, and immunofluorescence (Supplemental Figure 1).

Giantin- or GM130-knockdown HSG cells were established by lentiviral delivery of shRNAs using a pool of 3 different shRNA plasmids targeting Giantin (sh-Giantin) or GM130 (sh-GM130) mRNA (Santa Cruz Biotechnology Inc.) (Supplemental Table 2). The control consisted of a shRNA plasmid-A encoding a scrambled shRNA sequence that does not produce specific degradation of any known cellular mRNA (sh-control) (Santa Cruz Biotechnology Inc.). Lentiviral particles were generated by cotransfection of HEK293 cells with pLKO.1-shRNA (0.5 μg), VSV-g (0.1 μg), and PD8.9 (0.5 μg) constructs with Effectene (Qiagen). Forty-eight hours after transfection, the medium containing lentiviral particles was transferred to HSG cells. After 24 hours, cells were selected with 3 μg/mL puromycin and maintained with this drug every 2 weeks to obtain cell lines stably expressing the shRNAs. To evaluate the silencing efficiency of the studied golgins, we analyzed Giantin and GM130 transcript and protein levels and their localization (Supplemental Figure 2).

### RNA extraction and real-time PCR.

The RNA was extracted with yields and purity evaluated as previously described (58). One microgram of total RNA was reverse transcribed with oligo (dT), random primers, and the Superscript II enzyme (Invitrogen). Specific primers for Giantin, GM130, GMAP210, p115, GCP60, TFE3, CREB3, GalNT1, C1GalT, GCNT1, and h18S genes were designed with the AmplifiX 1.4 software (Supplemental Table 3). Real-time PCR was performed using 5X Hot FirePol EvaGreen qPCR Mix Plus (Solis Biodyne) with an AriaMx thermocycler (Agilent). Each specific transcript tested was expressed as its ratio to h18S, using the efficiency-calibrated model (62).

### Western blotting and IP assays.

The LSG biopsies or HSG cells were homogenized using RIPA buffer containing the Complete Protease Inhibitor Cocktail (Roche). Proteins were quantified using the bicinchoninic acid (BCA) protein assay (Pierce, Thermo Fisher Scientific), separated by SDS-PAGE under reducing conditions, and transferred to nitrocellulose membranes. Blots were then blocked with 5% milk and probed with specific primary antibodies (Supplemental Table 4) and horseradish peroxidase–conjugated secondary antibodies. Next, target proteins were detected by chemiluminescence, quantified by densitometry, and normalized to β-actin.

A pool of proteins extracted from LSGs of patients with SjD or individuals acting as controls was used for IP assays. The LSG or HSG cell extracts were prepared in a buffer containing 20 mM Tris-HCl pH 7.4, 150 mM NaCl, 1% Igepal, and protease inhibitors. Supernatants obtained after centrifugation were used for IP assays (500 μg of total protein per assay) with antibody-coated Protein-A/G-Sepharose beads. The negative control (irrelevant IgG) for all assays was an anti-integrin α6 antibody (Supplemental Table 4).

### Glycan analysis.

The LSG biopsies were homogenized using RIPA buffer containing the Complete Protease Inhibitor Cocktail (Roche). Proteins were separated by electrophoresis on polyacrylamide gels, transferred to PVDF membranes, and stained with Alcian Blue (8GX, Sigma-Aldrich). The stained bands were cut from the membrane, and the MUC5B-associated oligosaccharides were released by reductive β-elimination. The oligosaccharide mixtures were analyzed by high-performance anion exchange chromatography (HPAEC) using an ICS-3000 Pulsed Amperometric Detection (PAD) system with an electrochemical gold electrode, a CarboPac PA20, a 3 × 150-mm analytical column, and a CarboPac PA203 × 30-mm guard column (Dionex, Thermo Fisher Scientific). The oligosaccharides were also analyzed by mass spectrometry using an Ultraflex II matrix-assisted laser desorption ionization time of flight/time of flight (UV-MALDI-TOF/TOF) instrument (Bruker Daltonics). Samples were analyzed in positive ion mode using 2,5-dihydroxybenzoic acid as matrix.

### Immunofluorescence and immunohistochemistry.

For immunofluorescence, LSG were fixed in 1% paraformaldehyde (PFA) or Bouin’s solution for 6 hours and embedded in paraffin. Tissue sections were deparaffinized, rehydrated, and antigen recovery was performed in citrate buffer (pH 6.0) at 92°C for 25 minutes. Additionally, HSG cells cultured on glass coverslips were fixed in 2% PFA or cold acetone (−20°C) and permeabilized with 0.1% Triton X-100. The LSG sections or HSG cells were then blocked with 0.25% casein or 1% bovine serum albumin and sequentially incubated with specific primary and Alexa Fluor 488– or 546–conjugated secondary antibodies (Supplemental Table 4). Hoechst 33342 was used for nuclear staining. For immunohistochemistry, the LSG were fixed in Bouin’s solution for 6 hours and embedded in paraffin. Serial tissue sections were deparaffinized, rehydrated, and antigen recovery was performed in citrate buffer (pH 6.0) at 92°C for 5 minutes. The sections were treated with Bloxall blocking solution (Vector Laboratories) and then incubated with specific primary antibodies (Supplemental Table 4) and biotinylated secondary antibodies, followed by Vectastain ABC avidin peroxidase reagent (Vector Laboratories). Staining was revealed with ImmPACT DAB chromogen (Vector Laboratories), and nuclei were counterstained with Mayer’s hematoxylin. Rabbit or mouse IgG fractions were employed as a negative control. Immunohistochemistry images were captured under an Axiostar Plus light microscope (Zeiss). The total number of mucosal acini (PANH2 and EU-MUC5Bb positive) and the number of Sulfo-Lewis–positive (F2-positive) acini were quantified by two independent observers.

### Quantitative fluorescence analysis.

Fluorescent images were obtained in a C2 confocal laser scanning microscope (Nikon). For each analyzed protein, the pinhole diameter, laser intensity, and sensitivity were conserved in LSG sections from patients with SjD, individuals acting as controls, and different cell culture conditions. The images were deconvolved with the Huygens Pro 4.3.0 software, and the colocalization analysis was performed with the Imaris Classic 7.2.3 software to obtain the Manders coefficients M1/2. Fluorescence intensity was determined using the ImageJ software (https://imagej.net/; NIH).

### Transmission electron microscopy.

The LSG were fixed in 2.5% glutaraldehyde for 16 hours, rinsed in sodium cacodylate buffer pH 7.0, and postfixed in OsO_4_ for 90 minutes. The samples were stained in 1% uranyl acetate, dehydrated in acetone, and embedded in Epon. The sections were obtained using a Leica Ultracut R ultramicrotome, stained with uranyl acetate and lead citrate, and observed in a Tecnai transmission electron microscope (Philips). The area of the Golgi apparatus stacks and the luminal width of Golgi apparatus cisternae were quantified with ImageJ (NIH), as previously described (63).

### Statistics.

The statistical significance of differences between groups was analyzed with GraphPad Software, using the Mann-Whitney test. The Spearman’s rank correlation analysis was also performed. *P* values of less than 0.05 were considered significant.

### Study approval.

This study was approved by the Ethical Committee of the Facultad de Medicina of the Universidad de Chile (no. 154-2017). Written consent was obtained according to the Declaration of Helsinki prior to participation.

### Data availability.

The data sets shown in all the figures are provided in the Supporting Data Values file.

## Author contributions

MN, PC, and IC conceived the research study. MN, PC, and IC designed the experiments. MN, PC, MJB, SM, AC, ML, GB, KP, SI, LF, and IC performed the experiments. MN, PC, MJB, SM, AC, ML, GB, KP, SI, LF, CM, MJG, and IC analyzed and interpreted the data. SA, SM, AC, ML, GB, SG, CM, and MJG provided technical support and consultations. SA, SG, and CM collected the clinical samples. MN, PC, and IC wrote the manuscript. MN, PC, SA, MJB, SM, AC, ML, GB, SG, CM, KP, SI, LF, MJG, and IC critically revised, read, and approved the submitted version. The first author order was determined by flipping a coin.

## Supplementary Material

Supplemental data

Supplemental video 1

Supporting data values

## Version 1 10/10/2024

In-Press Preview

## Version 2 11/22/2024

Electronic publication
