# Supplementary material for: Giantin mediates Golgi localization of Gal3-O-sulfotransferases and affects salivary mucin sulfation in patients with Sjögren’s disease
Source: JCI Insight. 2024 Nov 22;9(22):e171585. doi: 10.1172/jci.insight.171585 (PMC11601944; doi:10.1172/jci.insight.171585)
Supplement: Supplemental data [file jciinsight-9-171585-s199.pdf]

Giantin mediates Golgi localization of Gal3-O-sulfotransferases and affects salivary mucin sulfation in Sjögren's disease patients

Supplemental Video 1: Altered Gal3ST-4 localization in sh-Giantin cells. Z-stack series of images showing Gal3ST-4 (green), GMAP120 (red), and nuclei (blue) in sh-Giantin cells fixed with cold acetone. Hoechst 33342 (blue) was used for nuclear staining.

https://youtube.com/shorts/4ZzVqkWGdG0?feature=share

Supplemental Table 1. Demographic and serological characteristics of control subjects, patients with Sjögren's disease and patients with Sarcoidosis

Parameters	Control subjects	Patients with Sjögren's Disease	Patients with Sarcoidosis
N° of individuals	24	24	5
Sex (female/male)	18/3	23/1	3/2
Age, mean (range), years	40 (21-61)	40 (20-68)	41 (26-62)
Focus score†			
1	0	7	0
2	0	7	0
≥ 3	0	10	0
USWSF, mL/15 min mean (range)	3.4 (0.3-7.5)	1.4 (0-5.8)	0.75 (0-1.8)
Positive serology			
Anti Ro N° (%)	0 (0 %)	22 (91.7 %)	0 (0 %)
Anti La N° (%)	0 (0 %)	16 (66.7 %)	0 (0 %)
ANA N° (%)	2 (0 %)	22 (91.7 %)	3 (60 %)
Rheumatoid factor (RF) N° (%)	0 (0 %)	12 (50 %)	1 (20 %)
ESSDAI, mean-median; IQR [25-75]	-	9.9-8 [5-17]	-

Patients with Sarcoidosis consulted the physician because of oral and/or ocular dryness symptoms and underwent salivary gland biopsy for suspected Sjögren's disease. The clinical diagnosis did not meet Sjögren's classification criteria and was sarcoidosis instead. All of them had a biopsy with non-caseating granulomas compatible with Sarcoidosis.

USWSF: Unstimulated whole salivary flow, N°: number, %: percentage, ESSDAI: EULAR Sjögren syndrome disease activity index; EULAR, European League against Rheumatism. †Number of foci/4 mm^2 of tissue. ANA: antinuclear antibodies. IQR, interquartile range.

Supplemental Table 2. sgRNA and shRNA sequences used to establish knockdown and knockout cell lines

**CRISPR-Cas9 sgRNA non-targeting control**		
	Sense: CACCGTAGGCGCGCCGCTCTCTAC	Antisense: AAACGTAGAGAGCGGCGCGCCTAC
**CRISPR-Cas9 sgRNA GOLGB1 Exon 8**		
	Sense: CACCGAACACGAAGAATCCTTGGT	Antisense: AAACACCAAGGATTCTTCGTGTTC
**GOLGB1 exon 8 primers for sequencing**		
	Sense: GGAGACTCTTCGTTGCTGCT	Antisense: TGACATTCTAGATAAACTAGGACCC
**Giantin shRNAs plasmids (h)**		
60685-SHA	Hairpin sequence: GATCCGAAGAACTCTCCAGAGTTATTCAAGAGATAACTCTGGAGAGTTCTTCTTTTT	
	Sense: GAAGAACUCUCCAGAGUUAtt	Antisense: UAACUCUGGAGAGUUCUUCtt
60685-SHB	Hairpin sequence: GATCCGCAAGAGGCTGATATTCAATTCAAGAGATTGAATATCAGCCTCTTGCTTTTT	
	Sense: GCAAGAGGCUGAUAUUCAAtt	Antisense: UUGAAUAUCAGCCUCUUGCtt
60685-SHC	Hairpin sequence: GATCCGAAGGTAGGTGAAATTGAATTCAAGAGATTCAATTTCACCTACCTTCTTTTT	
	Sense: GAAGGUAGGUGAAAUUGAAtt	Antisense: UUCAAUUUCACCUACCUUCtt
**GM130 shRNA plasmids (h)**		
41224-SHA	Hairpin sequence: GATCCCCAGCTATGTAACAAACAATTCAAGAGATTGTTTGTTACATAGCTGGTTTTT	
	Sense: CCAGCUAUGUAACAAACAAtt	Antisense: UUGUUUGUUACAUAGCUGGtt
41224-SHB	Hairpin sequence: GATCCGGAGTCGGTTAGACAACTATTCAAGAGATAGTTGTCTAACCGACTCCTTTTT	
	Sense: GGAGUCGGUUAGACAACUAtt	Antisense: UAGUUGUCUAACCGACUCCtt
41224-SHC	Hairpin sequence: GATCCGAAGATCACTGTCATCTAATTCAAGAGATTAGATGACAGTGATCTTCTTTTT	
	Sense: GAAGAUCACUGUCAUCUAAtt	Antisense: UUAGAUGACAGUGAUCUUCtt

All sequences are provided in 5′ → 3′ orientation
sgRNA: single guide RNA
shRNA: short-hairpin RNA

Supplemental Table 3. Primers used for RT-qPCR assays

Gene (encoded protein)	Accession number	Primer sequences
**GOLGB1 (Giantin)**	NM_001366282.2<br>NM_001366283.2<br>NM_001256486.2<br>NM_004487.5<br>NM_001366284.2<br>NM_001256487.2	F: 5’-GCAGGAAGCTGAGCAAGAAA-3’<br>R: 5’-TTCCTTCATGCTGGCATTGG
**GOLGA2 (GM130)**	NM_001366244.1<br>NM_001366246.1<br>NM_004486.5	F: 5’- AACATCGCTGCATCCAGCTT-3’<br>R: 5’- AATCTGCCATGCCACTCGTT-3’
**USO1 (P115)**	NM_001290049.1	F: 5’-AGGCTGCCAAGTAAACCAAG-3’<br>R: 5’-CTGGCCAGCTGAAACTGAAT-3’
**ACBD3 (GCP60)**	NM_022735.4	F: 5’-CACACTGACAGCTCCGAAAA-3’<br>R: 5’-GCCCACTGTAATCACGGAAT-3’
**TFE3 (TFE3)**	NM_001282142.2<br>NM_006521.6	F: 5’-AACAGCCCAGCACAGGTATT-3’<br>R: 5’-AAGAGCAAGGTTGGCTCAGA-3’
**CREB3 (CREB3)**	NM_006368.5	F: 5’-AACTCCAGGCCATGGTGATT-3’<br>R: 5’- GCTGTCTTTCGGCACTTCTG-3’
**GalNT1 (GalNT1)**	NM_001384438.1<br>NM_001384439.1<br>NM_001384440.1<br>NM_001384441.1<br>NM_001384442.1<br>NM_001384443.1<br>NM_001384444.1<br>NM_001384445.1<br>NM_001384446.1<br>NM_020474.4	F: 5’-TGGCCCAGTTACAATGCTCA-3’<br>R: 5’-ACCGACTTCCATTGCAGTCT-3’
**C1GalT1 (C1GalT1)**	NM_020156.5	F: 5’-TCACACTTGCCCAGGATGTT-3’<br>5’-ACAAGTTCCCAGGTGACGTT-3’
**GCNT1 (GCNT1)**	NM_001097633.2<br>NM_001097634.1<br>NM_001097635.2<br>NM_001097636.2<br>NM_001490.5	5’-TGTCACCTGGAATCAGCACT-3’<br>5’-CAGCAACGTCCTCAGCATTT-3’
**h18S**	NM_022551.2	F: 5’-GATATGCTCATGTGGTGTTG-3’<br>R: 5’-AATCTTCTTCAGTCGCTCCA-3’

F: forward; R: reverse

Supplemental Table 4. Antibodies used for Western blot and immunofluorescence

Primary antibodies	Host species	Immunogen	Source	Incubation dilution, time, T°
Anti-Giantin, polyclonal (PRB-114C), IgG1	rabbit	1-469 aa at the N-terminus of Giantin	BioLegend	WB: 1:5000, 20 h, 4°C<br>IF: 1:5000, 20 h, 4°C<br>IP: 1 µL, 15 h, 4°C
Anti-Giantin monoclonal, (ab37266), IgG1	mouse	Rat Giantin	Abcam	IF: 1:100, 20 h, 4°C
Anti-GM130, monoclonal (610822), IgG1	mouse	869-982 aa of rat GM130	BD Bioscience	WB: 1:1000, 20 h, 4°C<br>IF: 1:50, 20 h, 4°C<br>IP: 2 µL, 15 h, 4°C
Anti-GMAP210, monoclonal, (611712), IgG1	mouse	159-365 aa of human GMAP210	BD Transduction Laboratories	WB: 1:1000, 20 h, 4°C<br>IF: 1:50, 20 h, 4°C
Anti-PDIA1, monoclonal (1D3), IgG1	mouse	Peptide mapped at the end C-terminus of rat PDIA1	Enzo Life Sciences	IF: 1:300, 20 h, 4°C
Anti-TGN46, polyclonal (AHP500), IgG1	sheep	Recombinant human TGN46	AbDSerotec	IF: 1:250, 20 h, 4°C
Anti-PAPST1, polyclonal, IgG	rabbit	KAVPTEPPVQKV sequence of mouse PAPST1	Dr. Shoko Nishihara	IF: 1:500, 20 h, 4°C
Anti-GOLPH3, polyclonal (ab98023), IgG	rabbit	1-100 aa of human GOLPH3	Abcam	IF: 1:50, 20 h, 4°C
Anti-MUC1, monoclonal (M8), IgG1	mouse	DTR epitope of MUC1 VNTR	Dr. Dallas Swallow	IF: 1:50, 20 h, 4°C
Anti-MUC7, monoclonal (PANH3), IgG1	mouse	CRPKLPPSPNKP PKFPNPHQP sequence of MUC7 N-terminus	Dr. Ulla Mandel	IF: 1:50, 20 h, 4°C
Anti-Gal3ST2, polyclonal (orb183825), IgG	rabbit	KLH-conjugated peptide (33178 aa) of human Gal3ST2	Biorbyt	IF: 1:50, 20 h, 4°C
Anti-Gal3ST2, (H00079690-B01), IgG, polyclonal	rabbit	KLH-conjugated CELGPRRLRGEV ERL peptide	GenScript	WB: 1:1000, 20 h, 4°C<br>IP: 1 µL, 15 h, 4°C
Anti-Gal3ST4, polyclonal	rabbit	KLH-conjugated peptide (411-476 aa) of human Gal3ST4	Biorbyt	WB: 1:1000, 20 h, 4°C<br>IF: 1:25, 20 h, 4°C<br>IP: 4 µL, 15 h, 4°C
Anti-GalNAcT1, monoclonal (4D8), IgG1	mouse	41-559 aa of GalNAcT1	Dra. Ulla Mandel	WB: 1:10, 20 h, 4°C
Anti-Core2 GlcNAc-T2, polyclonal (ab227972), IgG	rabbit	Recombinant fragment of Core2 GlcNAc-T2	Abcam	IP: 2 µL, 15 h, 4°C
Anti-Integrin α6, monoclonal (BQ16), IgG, sc-13542	mouse	Human Integrin α6	Santa Cruz Biotechnology	IP: 1 µL, 15 h, 4°C

Anti-MUC5B, monoclonal (clone PANH2), IgG1	mouse	Partially deglycosylated MUC5B	Dra. Ulla Mandel	IHQ: non-diluted, 20 h, 4°C
Anti-MUC5B, monoclonal (EU-MUC5Bb), IgG1	mouse	RNREQVGKFKMC sequence on CYS domains of MUC5B	Dra. Dallas Swallow	IHQ: 1:250, 20 h, 4°C
Anti-sulfo-Lewis a and c, monoclonal (F2), IgM	mouse	sulfo-Lewis a and c	Dr. Enno Veerman	IHQ: 1:50, 20 h, 4°C
Anti-GRP78 (BiP), polyclonal (ab21685), IgG	rabbit	proprietary information	Abcam	IF: 1:250, 20 h, 4°C
Anti-β-actin, monoclonal (clone C4)	mouse	Actin of chicken gizzard	MP Biomedicals	WB: 1:15.000, 1 h, RT

**Secondary antibodies**	**Host species**	**Source**	**Dilution**
Anti-Rabbit IgG (H+L) Cross-Adsorbed, HRP conjugated	goat	Pierce® by Thermo Scientific, 31462	WB: 1:10.000
Anti-Mouse IgG (H+L) Cross-Adsorbed, HRP conjugated	goat	Pierce® by Thermo Scientific, 31432	WB: 1:10.000
Anti-Rabbit IgG (H+L) Alexa Fluor®488/546 conjugated	goat	Invitrogen by Thermo Fisher Scientific, A-11008 (488) and A-11010 (546)	IF: 1:200-1:100
Anti-Mouse IgG (H+L) Alexa Fluor® 488/546 conjugated	goat	Invitrogen by Thermo Fisher Scientific, A-11001 (488) and A 11003 (546)	IF: 1:200-1:100
Anti-Mouse/Rabbit IgG Biotinylated	horse	Vector Laboratories Vectastain Elite ABC Universal Plus Kit PK-8200	IHQ: R.T.U.

aa: aminoacids; KLH: keyhole limpet hemocyanin; HRP: horseradish peroxidase, RT: room temperature, R.T.U: ready to use

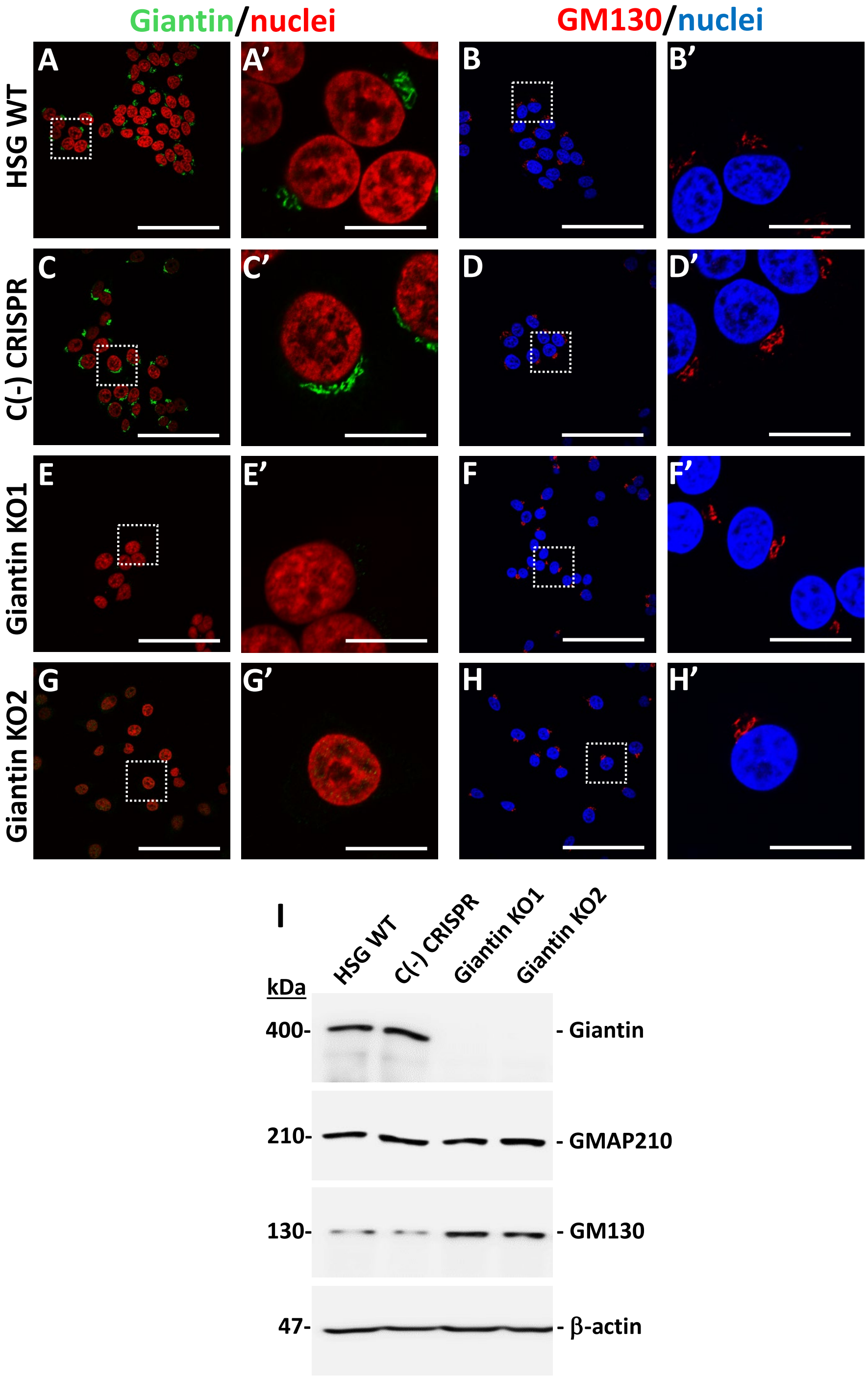

Supplemental Figure S1. Establishment of CRISPR/Cas9 Giantin Knockout (KO) cell lines. Representative micrographs of wild-type (WT) HSG cells (**A-B**), negative control (C(-)) CRISPR/Cas9 HSG cells (**C-D**), and two different cell lines of CRISPR/Cas9 Giantin KO cells (**E-H**). A, C, E, and G show Giantin (green) and nuclei (red). A’, C’, E’, and G’ are higher magnifications of regions surrounded by dashed lines in A, C, E, and G. B, D, F, and H show GM130 (red) and nuclei (blue). B’, D’, F’, and H’ are higher magnifications of regions surrounded by dashed lines in B, D, F, and H. Hoechst 33342 (blue) was used for nuclear staining. Bars: 10 µm. **I,** Representative WB of Giantin, GMAP210, and GM130 in WT HSG cells, C(-) CRISPR/Cas9 HSG cells, and two different cell lines of CRISPR/Cas9 Giantin KO cells. β-actin was used as a loading control**.**

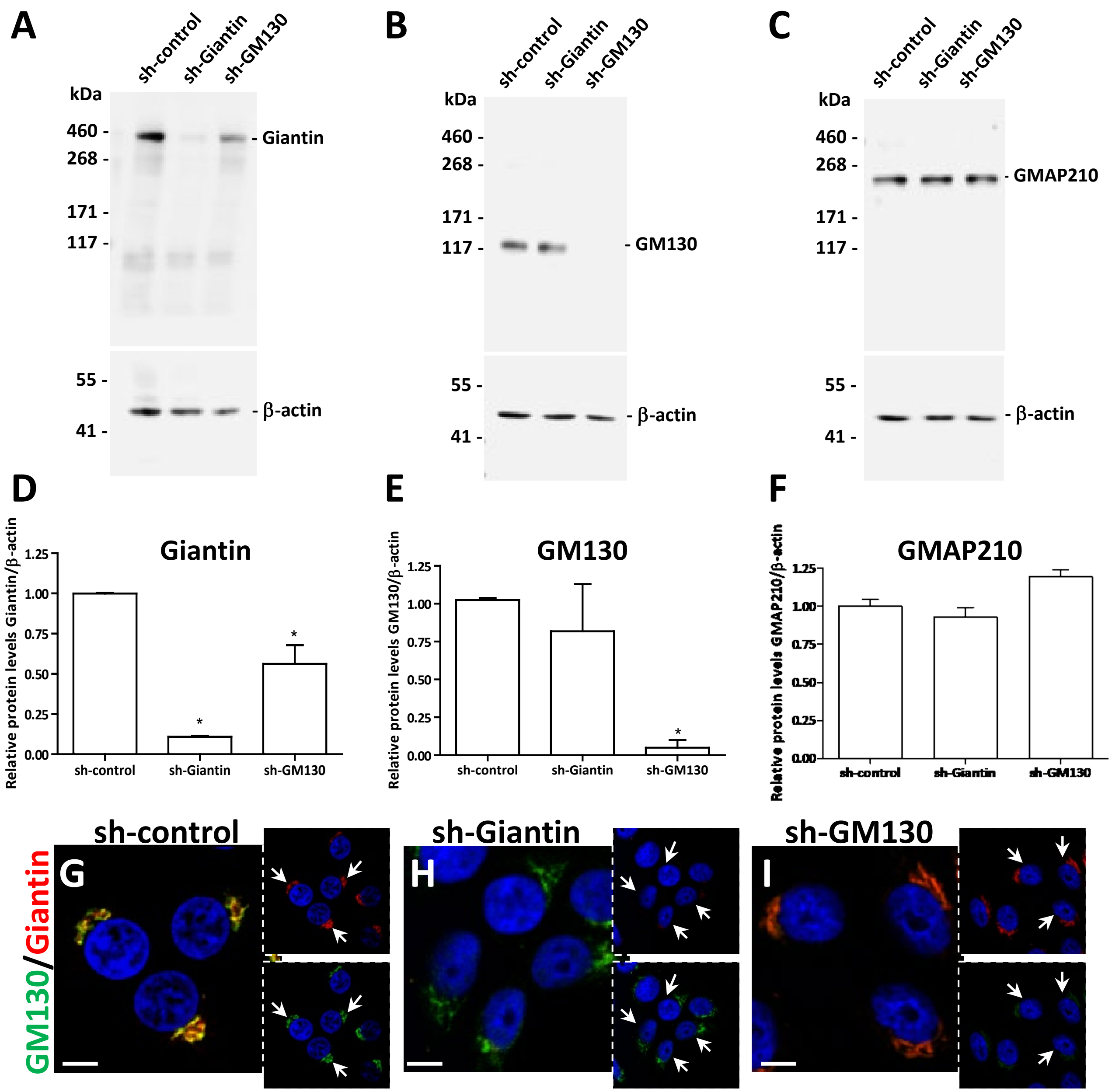

Supplemental Figure S2. Establishment of Giantin or GM130 knockdown cells. A-F, Representative WB of Giantin (A), GM130 (B), and GMAP210 (C) in sh-control, sh-Giantin, and sh-GM130 cells. β-actin was used as a loading control. **G-I**, Representative images of Giantin (green) and GM130 (red) detection by immunofluorescence in sh-control (G), sh-Giantin (H), and sh-GM130 (I) cells. Bars: 10 µm. (*) p values ≤0.05 were considered significant.

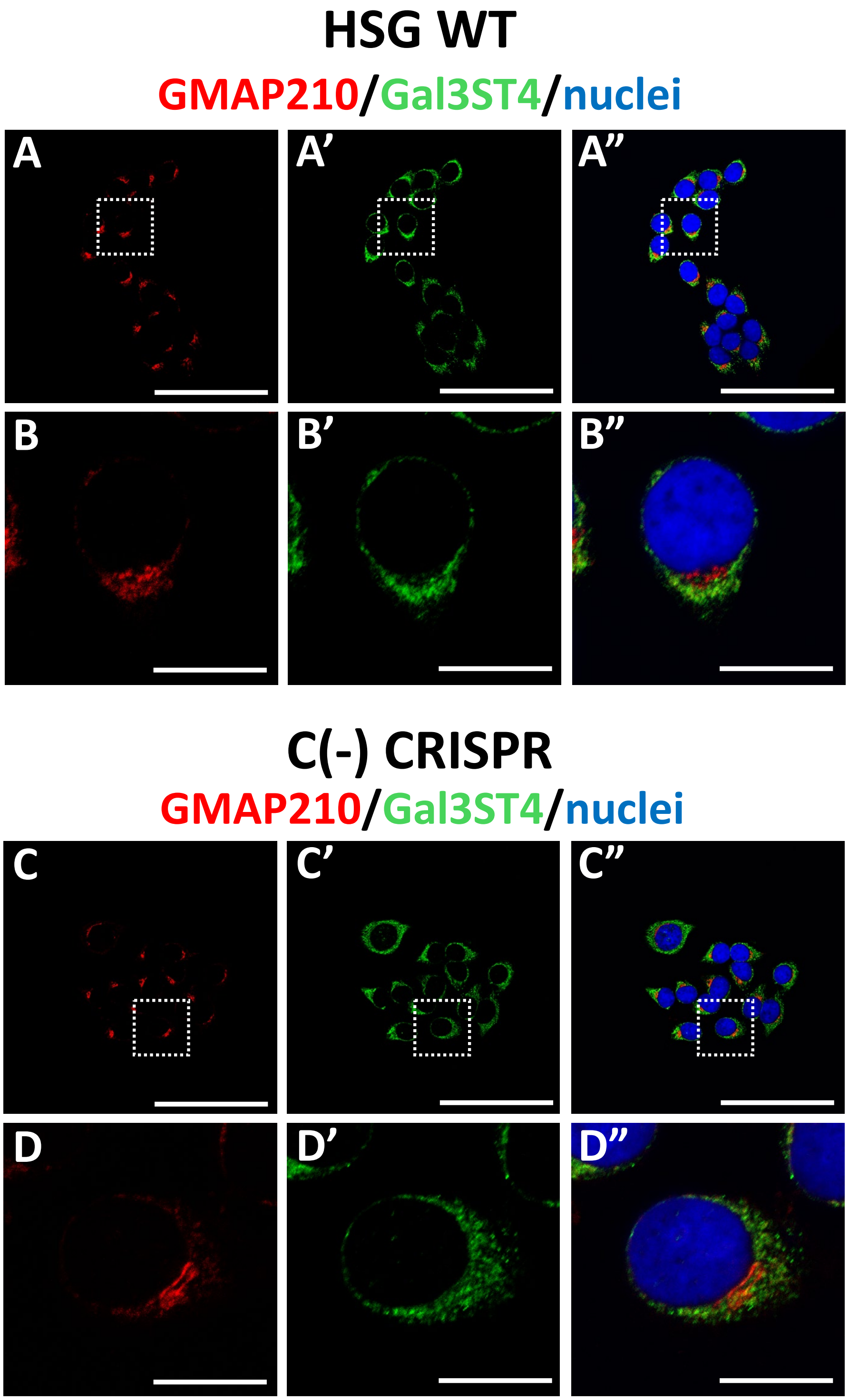

Supplemental Figure S3. Localization of Gal3ST-4 in HSG cells expressing Giantin. Representative micrographs of the double staining of Gal3ST-4 (green) and GMAP210 (red) in wild-type (WT) HSG cells (**A-B**) and negative control (C(-)) CRISPR/Cas9 HSG cells (**C-D**), B and D are higher magnifications of the regions enclosed by white dashed lines in A and C. Hoechst 33342 (blue) was used for nuclear staining. Bars A and C: 50 μm; B and F: 10 μm.

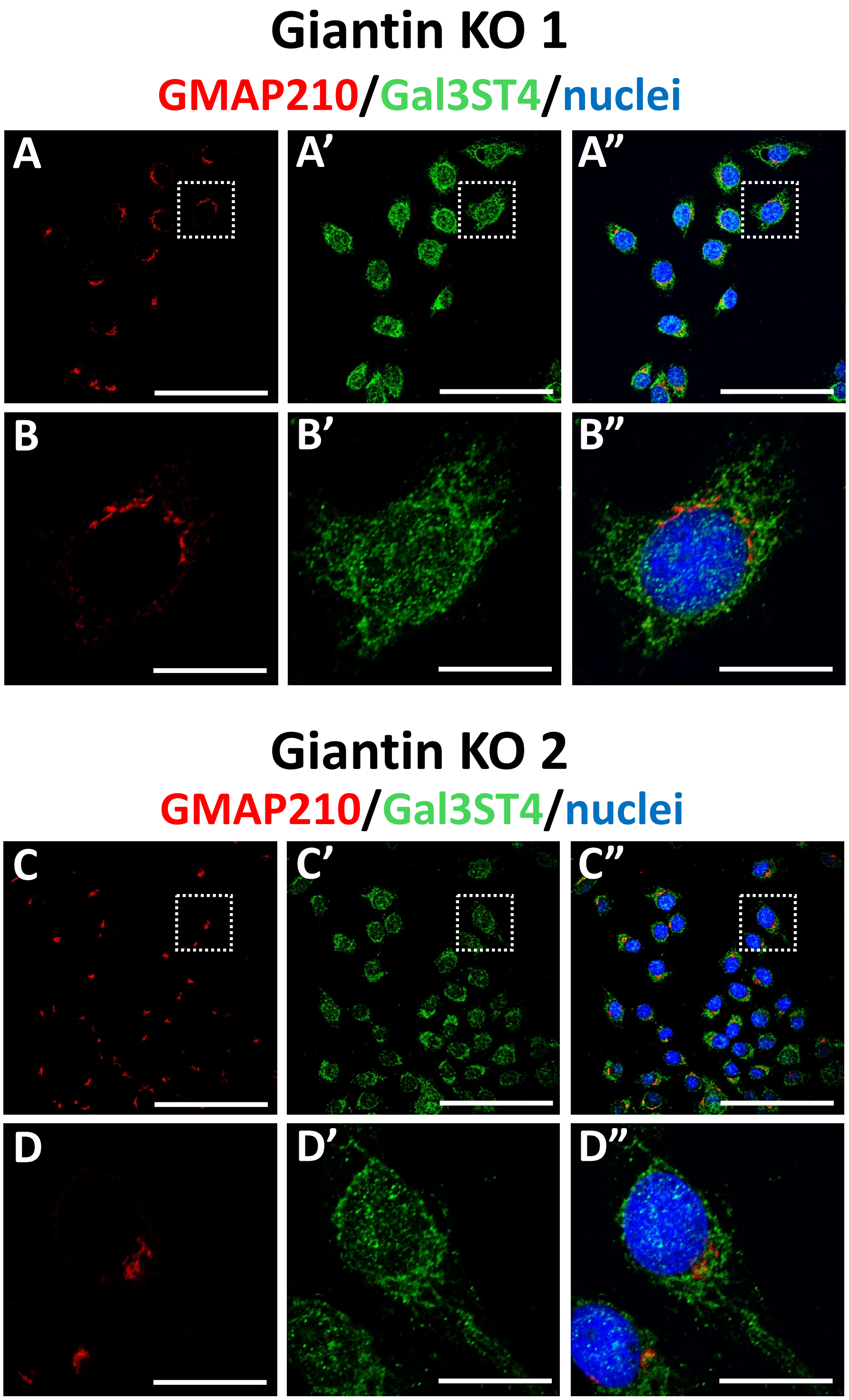

Supplemental Figure S4. Altered localization of Gal3ST-4 in CRISPR/Cas9 Giantin knockout (KO) cells. Representative micrographs of the double staining of Gal3ST-4 (green) and GMAP210 (red) in two cell lines: KO 1 (**A-B**) and KO 2 **(C-D)** of CRISPR/Cas9 Giantin KO cells. B and D are higher magnifications of the regions enclosed by white dashed lines in A and C. Hoechst 33342 (blue) was used for nuclear staining. Bars A and C: 50 µm; B and F: 10 µm.

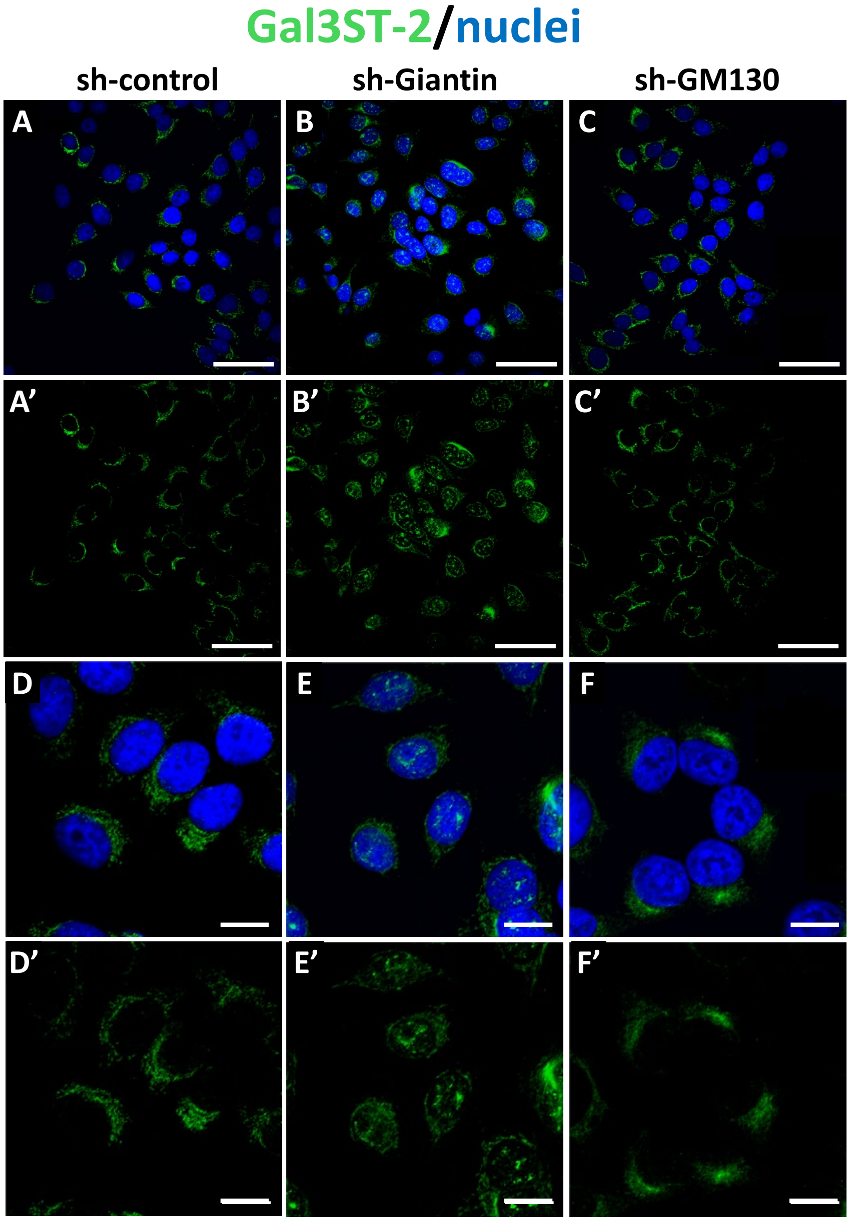

Supplemental Figure S5. Altered Gal3ST-2 localization in sh-Giantin cells. Representative micrographs of Gal3ST-2 (green) staining and nuclei (blue) in HSG cells. **A**, In most sh-control cells, Gal3ST-2 is located adjacent to the nucleus in a “crescent moon shaped” distribution; in a smaller percentage of cells, it is also located in close proximity to the nucleus, although showing a ring-shaped distribution. **B**, In sh-Giantin cells, this adjacent location is maintained, although a mark is also apparently observed “on the nucleus.” **C**, the distribution of Gal3ST-2 in sh-GM130 cells is quite similar to that of sh-control cells. **D-F**, show cells at higher magnification. Hoechst 33342 (blue) was used for nuclear staining. Bars A-C: 50 μm and D-F: 10 μm.

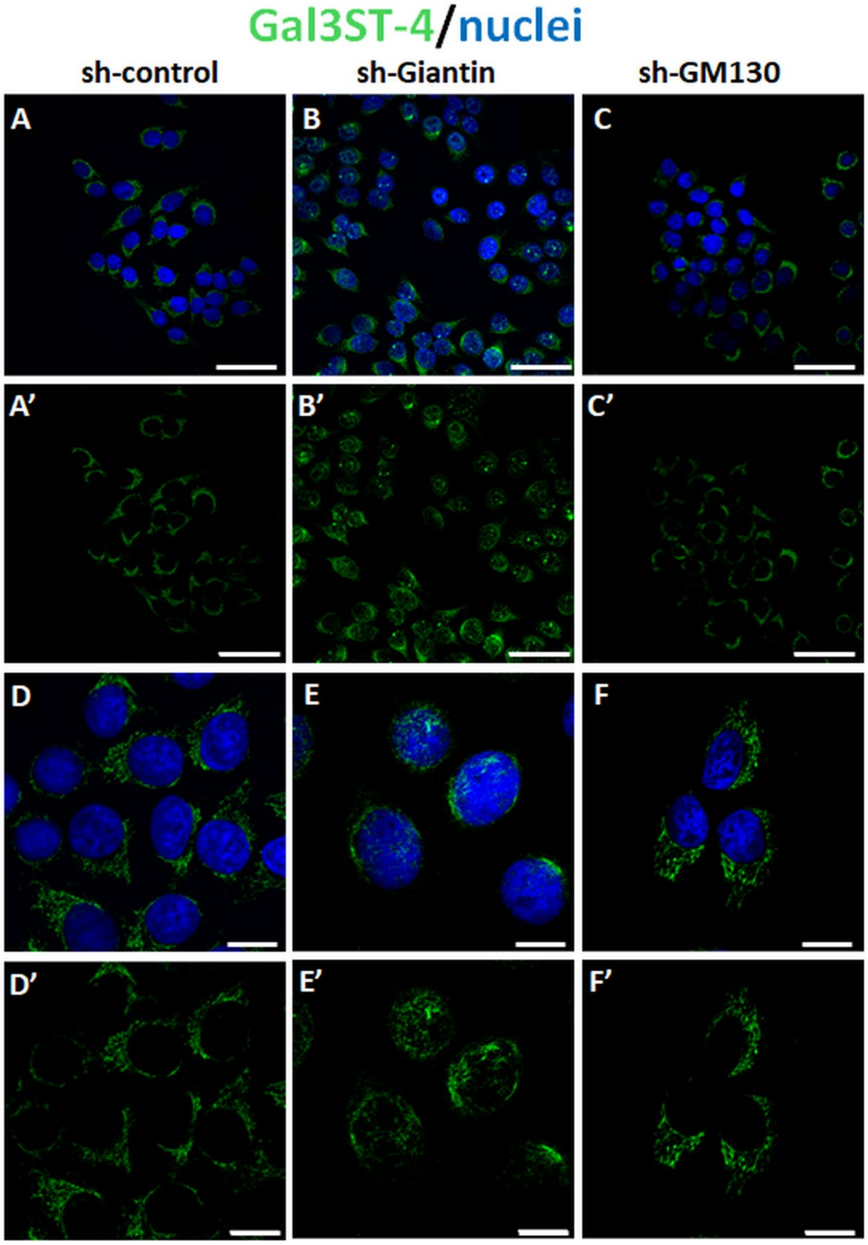

Supplemental Figure S6. Altered Gal3ST-4 localization in sh-Giantin cells. Representative micrographs of Gal3ST-4 (green) staining and nuclei (blue) in HSG cells. **A,** In most sh-control cells, Gal3ST-4 is located adjacent to the nucleus in a "crescent moon shaped" distribution; in a smaller percentage of cells, it is also located adjacent to the nucleus, although in a ring-shaped distribution. **B,** In sh-Giantin cells, this adjacent location is maintained, although a mark is also observed, apparently, "on the nucleus." **C**, The distribution of Gal3ST-4 in sh-GM130 cells is quite similar to that of sh-control cells. **D-F**, show cells at higher magnification. Hoechst 33342 (blue) was used for nuclear staining. Bars A-C: 50 μm and D-F: 10 μm.

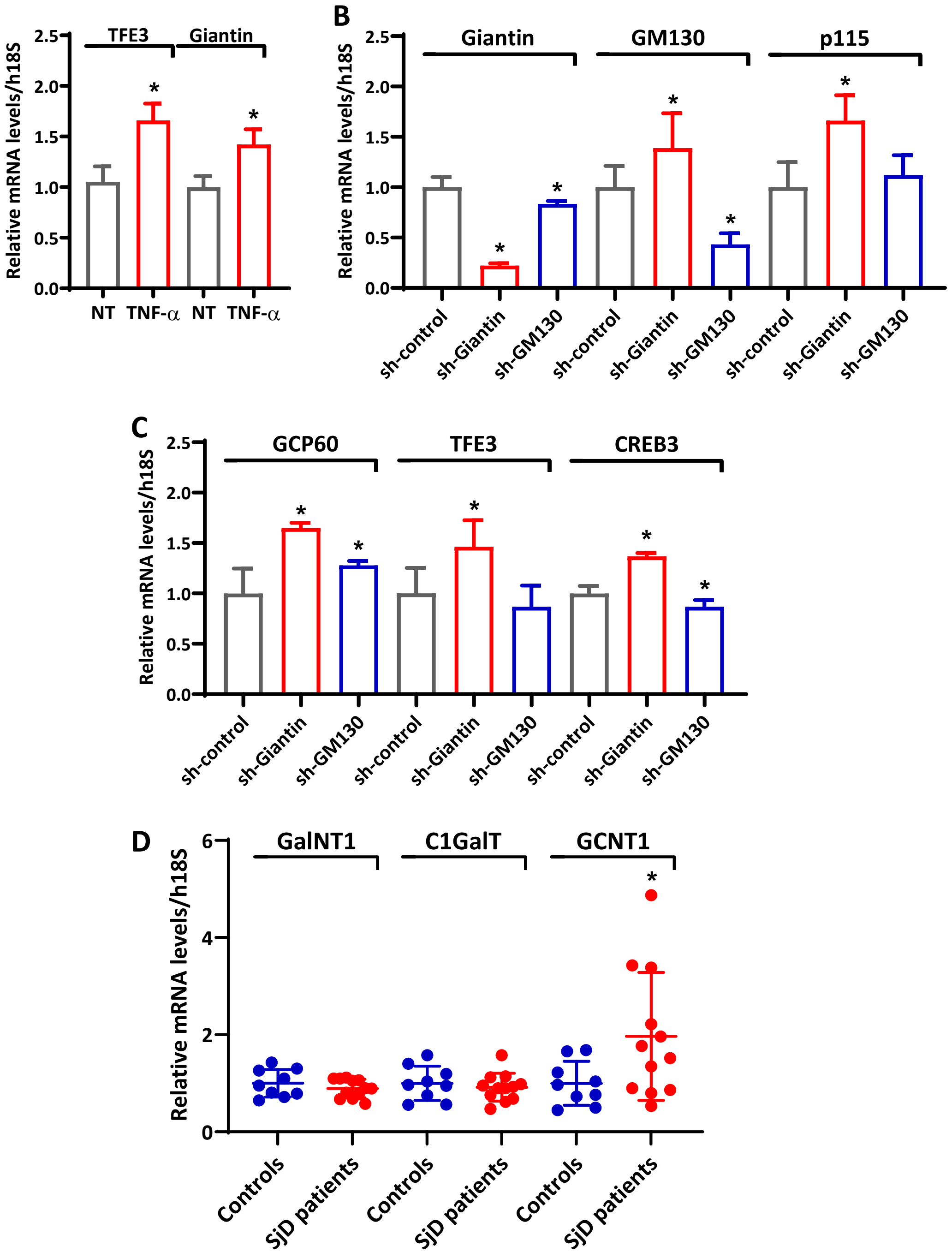

Supplemental Figure S7. Increased mRNA levels of key components of the Golgi stress response. A, Relative TFE3 and Giantin mRNA levels in non-treated (NT) HSG cells and HSG cells stimulated with 10 ng/mL TNF-α for 24 h. **B-C,** Relative mRNA levels of Giantin, GM130, p115, GCP60, TFE3, and CREB3 in sh-control, sh-Giantin, and sh-GM130 cells. **D,** Relative GalNT1, C1GalT, and GCNT1 mRNA levels in LSG from controls and SjD patients. h18S was used as a housekeeping gene. (*) p values ≤0.05 were considered significant.

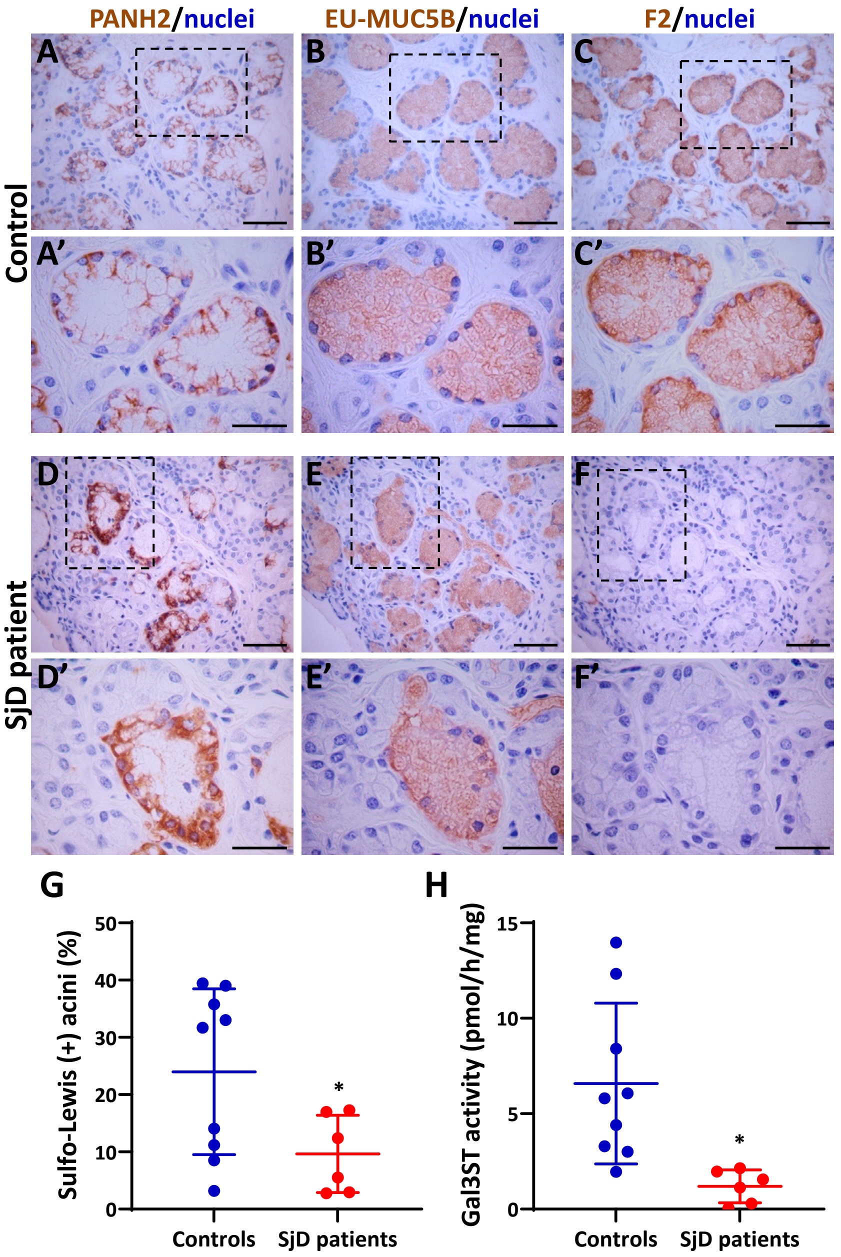

Supplemental Figure S8. Decreased sulfo-Lewis-positive acini in LSG from SjD patients. Representative images of immunohistochemical detection of partially glycosylated MUC5B (PANH2) (A and D), the MUC5B polypeptide backbone independent of its glycosylation status (EU-MUC5B) (B and E), and sulfo-Lewis a and c residues (F2) (C and F) in serial sections of LSG from controls (**A-C**) and SS patients (**D-F**). A'-F' higher magnifications of the acini surrounded by black dashed lines in A-F. Bars A-F: 100 μm and A'-F': 50 μm. **G**, Percentage of sulfo-Lewis a and c (F2)-positive acini in LSG from controls and SjD patients. **H**, Gal3STs activity levels in LSG from SjD patients and controls were determined in a previous study from our laboratory (Castro et al., 2012, DOI: 10.1093/rheumatology/ker351).

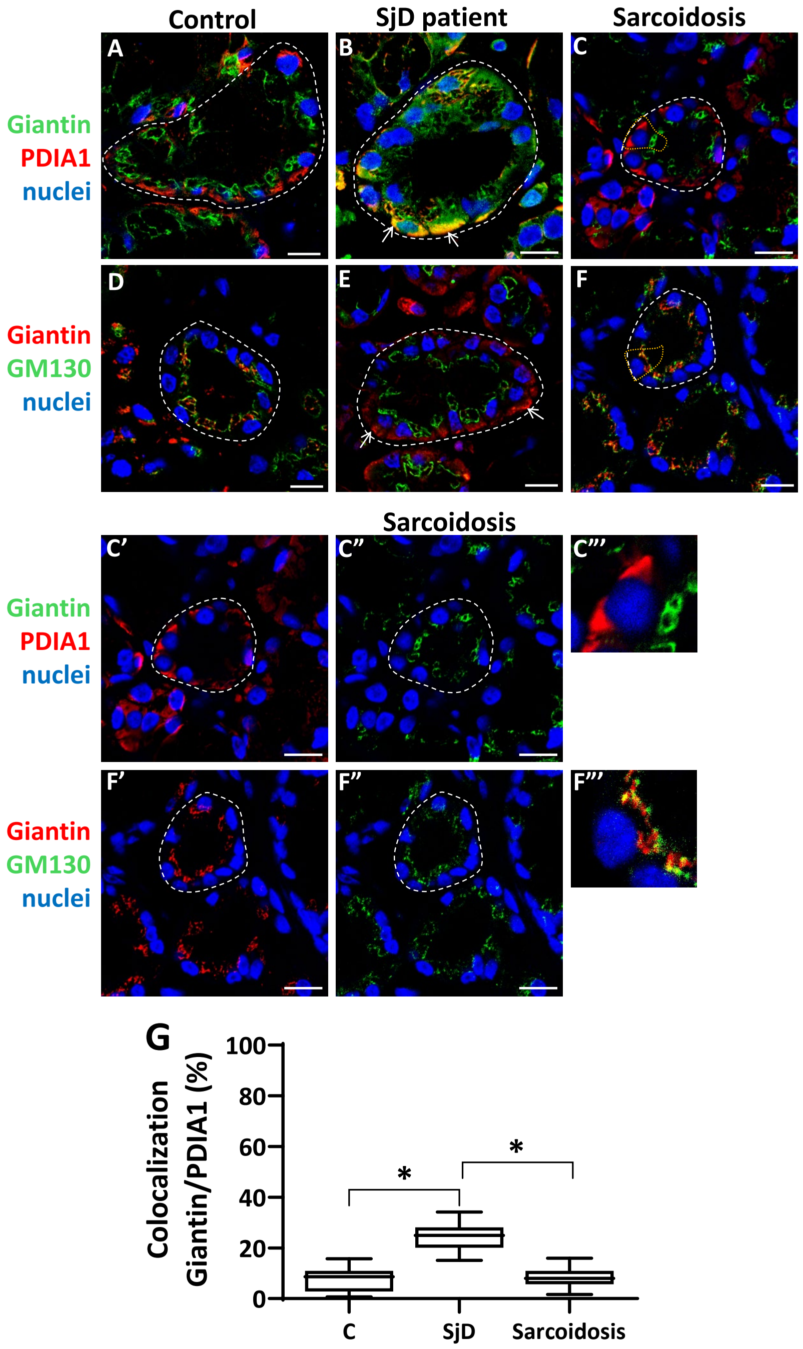

Supplemental Figure S9. Altered localization of Giantin in LSG from SjD patients, but not in LSG from patients with sarcoidosis. A-C, Representative micrographs of double staining of Giantin (green) and PDIA1 (red) in a LSG section from a control subject (A), a SjD patient (B), and a patient with sarcoidosis (C). **D-F**, Representative micrographs of double staining of Giantin (red) and GM130 (green) in a LSG section from a control subject (D), a SjD patient (E), and a patient with sarcoidosis (F). White arrows show Giantin staining in the basolateral region in SjD patients. Hoechst 33342 (blue) was used for nuclear staining. C', C", F', and F" show the green and red channels separately. C'" and F'" are higher magnifications of the acinar cell surrounded by yellow dashed lines in C and F. The white dashed lines surround the acinar boundaries. L: lumen and bars: 10 μm. **G**. Box plot showing the colocalization analysis of Giantin and PDIA1 in acini from LSG sections of 5 controls, 5 SjD patients, and 5 patients with sarcoidosis. Boxes represent the 25th to 75th percentiles; the lines within the boxes represent the median; and the whiskers represent the minimum and maximum. (*) p values < 0.05 were considered significant using the Mann-Whitney test.
